# Intraepithelial neutrophils in mammary, urinary and gall bladder infections

**DOI:** 10.1186/s13567-019-0676-5

**Published:** 2019-07-19

**Authors:** Dvir Mintz, Hagit Salamon, Michal Mintz, Ilan Rosenshine, Nahum Y. Shpigel

**Affiliations:** 1 The Koret School of Veterinary Medicine, The Hebrew University of Jerusalem, The Robert H. Smith Faculty of Agriculture, Food and Environment, POB 12, 76100 Rehovot, Israel; 2 Department of Microbiology and Molecular Genetics, IMRIC, Faculty of Medicine, The Hebrew University of Jerusalem, 91120 Jerusalem, Israel

## Abstract

Neutrophil mobilization is a crucial response to protect the host against invading microorganisms. Neutrophil recruitment and removal have to be tightly regulated to prevent uncontrolled inflammation and excessive release of their toxic content causing tissue damage and subsequent organ dysfunctions. We show here the presence of live and apoptotic neutrophils in the cytoplasm of inflamed mammary, urinary and gall bladder epithelial cells following infection with *E. coli* and Salmonella bacteria. The entry process commenced with adherence of transmigrated neutrophils to the apical membrane of inflamed epithelial cells. Next, nuclear rearrangement and elongation associated with extensive actin polymerization enabled neutrophils to crawl and invaginate the apical membrane into cytoplasmic double membrane compartments. Scission of the invaginated cell membrane from the entry point and loss of these surrounding membranes released intracellular neutrophils into the cytoplasm where they undergone apoptotic death. The co-occurrence of this observation with bacterial invasion and formation of intracellular bacterial communities (IBCs) might link entry of infected neutrophils to the formation of IBCs and chronic carriage in *E. coli* mastitis and cystitis and Salmonella cholecystitis.

**Electronic supplementary material:**

The online version of this article (10.1186/s13567-019-0676-5) contains supplementary material, which is available to authorized users.

## Introduction

Massive recruitment of blood neutrophils to sites of infection is the hallmark of many diseases [1]. Safe disposal and clearance of recruited neutrophils is essential to prevent host-tissue injury and progression of disease. This is achieved by three known mechanisms; (1) migration and mobilization to the lumen of epithelial-lined structures (e.g. urine, milk, bile, bronchi, gut) (2) reverse migration into blood, and (3) phagocytosis or efferocytosis by macrophages [2–4]. Resident and mobilized macrophages are frequently described as major contributors to phagocytosis and intracellular elimination of dying neutrophils while eliciting anti-inflammatory cytokines [5, 6]. This notion is based on many studies using peritonitis, air pouch or pneumonia mouse models, where abundant mobilization of blood monocytes or tissue macrophages is typically observed. However, in some tissues and disease processes macrophages may not be sufficient or even essential to fulfill this task. Current data suggest that in some mucosal surfaces or barrier epithelium, like urinary and gall bladder or the mammary alveoli, phagocytosis of apoptotic neutrophils by macrophage may not be the only or most important mechanism of neutrophil safe disposal and homeostasis of inflammation. We previously showed that in the mammary gland macrophage mobilization into the alveoli is sparse and cannot be a major mechanism for homeostasis of inflammation [7, 8]. Alternatively, recent studies demonstrated the role of neutrophils transmigrating across epithelial barriers in inflammatory regulation and resolution [9, 10]. Further to the role of epithelial cell in the development of inflammation and recruitment of neutrophils, various crosstalk mechanisms between these two cells are involved in the regulation and resolution of this potentially harmful process [11]. Such mechanisms include localized O_2_ depletion (“inflammatory hypoxia”), release of extracellular nucleotides and microvesicle-dependent exchange of microRNAs [12, 13]. In line with these intercellular communications, we discovered the presence of infected neutrophils in epithelial cells. Intracellular neutrophils were observed in the cytoplasm of epithelial cells where they undergo apoptotic death. This facet of neutrophil biology might be linked to safe disposal of neutrophils and regulation of inflammation in mastitis, urinary tract infection (UTI) and cholecystitis. These conditions are also associated with the formation of intracellular bacterial communities (IBC) in mammary, urinary and gall bladder epithelial cells [8, 14–18]. Our observation might implicate a link between neutrophil entry into epithelial cells and IBC formation in these tissues.

## Materials and methods

### Ethics statement

This study was carried out in strict accordance with the recommendations for the Care and Use of Laboratory Animals of the National Institute of Health. All procedures including animal studies were conducted following the guidelines for the Care and Use of Laboratory Animals of the Israel Ministry of Health, and in accordance with Israeli law. The protocol was approved by the Animal Care and Use Committee (IACUC) of the Hebrew University of Jerusalem. Institutional review board (IRB) and IACUC approvals were obtained prospectively (Ethics Committee for Animal Experimentation, Hebrew University of Jerusalem; MD-11-12692-4, MD-09-12140-3 and MD-15-14325-3).

### Mouse mastitis model

We examined by retrospective analysis mammary tissues obtained from *E. coli* (ECP4/pSA11-GFP) intramammary challenge studies in C3H/HeJ (TLR4-defective) and C57BL/6 TLR2-deficient mice that were previously described [7, 8, 14]. Similarly, lactating wild type C57BL/6 mice (Harlan Biotech, Rehovot Israel) were challenged with 10^1^ to 10^6^ CFUs of the non-pathogenic laboratory strain *E. coli* DH5α (Invitrogen).

### Mouse ascending cholecystitis model

5 to 7 weeks old C57BL/6 mice were anaesthetized using ketamine (75 mg/kg Ketamidor Richter Pharma) and medetomidine (1 mg/kg Dexdomitor Orion Corporation) mixture, and were laid in the dorsal recumbence position. Before surgery, carprofen (5 mg/kg, Rimadyl, Pfizer Animal health) was administered subcutaneously. The surgical area was shaved and depilated and the skin was scrubbed and disinfected with betadine and 70% (v/v) ethanol. A midline vertical incision was made through the skin and abdominal wall exposing the peritoneal cavity, and the cut edges were separated by an eye retractor to expose the gallbladder. A fine needle syringe was used to inject 10^5^ CFUs of *Salmonella enterica* serovar Typhimurium/P*oxyS*-*gfp* strain SL1344 [19] suspended in 10 µL PBS into the gallbladder. The abdomen was closed using standard surgical techniques. The whole gallbladder was removed 24 h post-infection, and prepared for microscopic analysis as described below.

### Mouse cystitis model

5 to 7 weeks old female C57BL/6 mice, were challenged with uropathogenic *E. coli* (UPEC) UTI89 serotype O18:K1:H7 [20] as previously described [21]. Mice were anaesthetized as described above, and were laid in the dorsal recumbence position. The external urinary ostium was than cleansed using 70% ethanol. Using fine tweezers, the external ostium was extended and a 24G IV catheter was inserted into the urethra. Micturition was induced through a mild lower abdomen massage, residual urine was aspirated and bacteria were introduced into the urinary bladder. The mouse was left to recover and was returned to its cage. The mice were euthanized 24 h post-infection, dissected in the midline to remove the whole urinary bladder for microscopic analysis as described below.

### Cell culture and neutrophil entry assays

Mouse mammary gland epithelial EpH4 cells and human urinary bladder 5637 cells (kindly donated by B. Aroeti, Hebrew University) were used for in vitro neutrophil entry assays. EpH4 cells were cultured and maintained in complete Dulbecco modified Eagle’s medium (DMEM) supplemented with 4 mM l-glutamine, 1% penicillin/streptomycin, 10% heat-inactivated fetal bovine serum (FBS) and 25 mM HEPES (Biological Industries, Kibbutz Beit Haemek, Israel), in a 5% CO_2_ humidified incubator at 37 °C. The 5637 cells were cultured and maintained in complete Roswell Park Memorial Institute medium (RPMI) 1640 supplemented with 4 mM l-glutamine, 1% penicillin/streptomycin, 10% heat-inactivated FBS, 25 mM HEPES, 1 mM sodium pyruvate and 2.5 g/L glucose (all ingredients except glucose—Biological Industries, Kibbutz Beit Haemek, Israel. Glucose—Sigma Aldrich, Israel), in a 5% CO_2_ humidified incubator at 37 °C.

Bovine polymorphonuclear cells (PMN) were isolated as described previously [22]. In brief, whole blood was extracted from healthy cows into heparinized syringes and PMN were isolated by multiple centrifugation and RBC lysis steps. For the PMN entry assay, 10^5^ EPH4 or 5637 cells were seeded onto sterilized 13 mm cover slips in a 24-well plate, and were cultured in 2 mL of their appropriate medium for 7 days prior to the assay. On the day of the assay, culture medium was discarded and 10^4^ PMN (fresh, apoptotic or fixed) were added to each well in fresh medium with no FBS supplemented with 1% autologous bovine serum, and were co-cultured with the cells for 12–18 h. The next morning the medium was removed and wells were washed twice with medium with no serum, then incubated without serum for 3–5 h. At the end of incubation medium was removed and cells were prepared for Diff-Quick staining, immunofluorescence staining, and TEM as described below.

### Preparation of fixed bovine neutrophils

Neutrophils extracted from bovine blood were centrifuged (700 *g*, 10 min, 4 °C) and suspended into a concentration of 2 × 10^6^ cells in 1 mL of 4% PFA for 10 min, than washed in 10 nM NH_4_Cl solution for 10 min, proceeded by another wash in PBS, and resuspended in complete medium containing autologous serum to the desired concentration.

### Apoptotic bovine neutrophil induction

Fresh neutrophils extracted from bovine blood were spread in a thin film on an open 90 mm plate under UV light for 7 min (using Stratagene Stratalinker 1800 UV Crosslinker). After the UV impulse, the cells were incubated to allow recovery in complete medium for 3 h, apoptotic status was decided by microscopic morphology, apoptotic neutrophils show condensation of nuclear material into one or more dark-stained nuclear bodies and by flow-cytometry (BD Accuri C6) using Annexin V FITC (BD 560931) and propidium iodide (PI) dye to differentiate the dead cells, before use (Additional file 9). CFlow software (BD) was used for flow cytometry analysis. All apoptotic cells used in the interaction/uptake experiments were greater than 70% apoptotic and more than 90% viable as judged by trypan blue exclusion and flow cytometry.

### Surface biotinylation

Epithelial monolayers with intraepithelial bovine neutrophils were prepared as described above. Culture medium was discarded, cells were washed with PBS, incubated with biotin (0.5 mg/mL; Thermo 21355) for 10 min at 37 °C, washed in PBS, fixed in 2% PFA for 15 min at room temperature, and stained with avidin-FITC (Sigma Immuno Chemical, A-2050) and DAPI.

### Histochemical analysis

Mammary samples for histological analysis were prepared as previously described [8, 14]. Briefly, paraffin sections were stained with haematoxylin and eosin (H&E) and cryosections were stained with phalloidin-TRITC (Sigma) to visualize filamentous actin (F-actin) and 4′,6′-diamidino-2-phenylindole (DAPI) (Invitrogen, Carlsbad, CA, USA) to visualize nuclear DNA.

Gall bladder and urinary bladder whole mounts were pinned onto a silicone pinning pad (SYLGARD 184 Dow Corning), fixed with 2.5% formaldehyde and stained with phalloidin-TRITC and DAPI or WGA Alexa Flour 633 (ThermoFisher W21404) and Sytox Orange (Invitrogen) as previously described [21].

Tissue sections were imaged with a Nikon Eclipse E400 epifluorescence microscope with an Olypus DP70 camera and images were merged using Adobe Photoshop software. Confocal images were acquired using a Leica TCS SP2 laser scanning spectrum confocal system (Leica Microsystems) and images were merged using Leica confocal software.

### Transmission electron microscopy

Samples for transmission electron microscopy (TEM) were fixed with a mixture of 2.5% glutaraldehyde in 0.1 M phosphate buffer, pH 7.2, for 2 h and then washed with 0.1 M phosphate buffer. After osmification, dehydration and embedding (Epon), the tissue was sectioned using an LKB-ultrotome 8800 III. Thick sections (1 μm) were stained with toluidine blue for light microscopy and thin sections (70 nm) were stained with uranyl acetate and Reynold’s lead citrate for transmission electron microscopy (TEM) and observed with a Tecnai 12 (Phillips, Eindhoven, The Netherlands) TEM equipped with MegaView II CCD camera and AnalySIS version 3.0 software.

### Statistical analysis

Parametric (% cell-in-cell; Additional file 9D) and non-parametric (CFU counts; Additional file 4A) data were calculated as the mean and median, respectively. For comparison of parametric and non-parametric data, t-test and non-parametric Mann–Whitney two-independent-samples test (respectively) were applied using GraphPad Prism 6 (GraphPad Software, Inc.). *P* value of 0.05 or less was considered significant. CFUs in challenge studies were analyzed using One-Sample Wilcoxon Signed Rank Test and the null hypothesis was median of CFUs equals to challenge dose.

## Results

### Bacterial communities in mammary urinary and gall bladder epithelia

**Figure 1:**
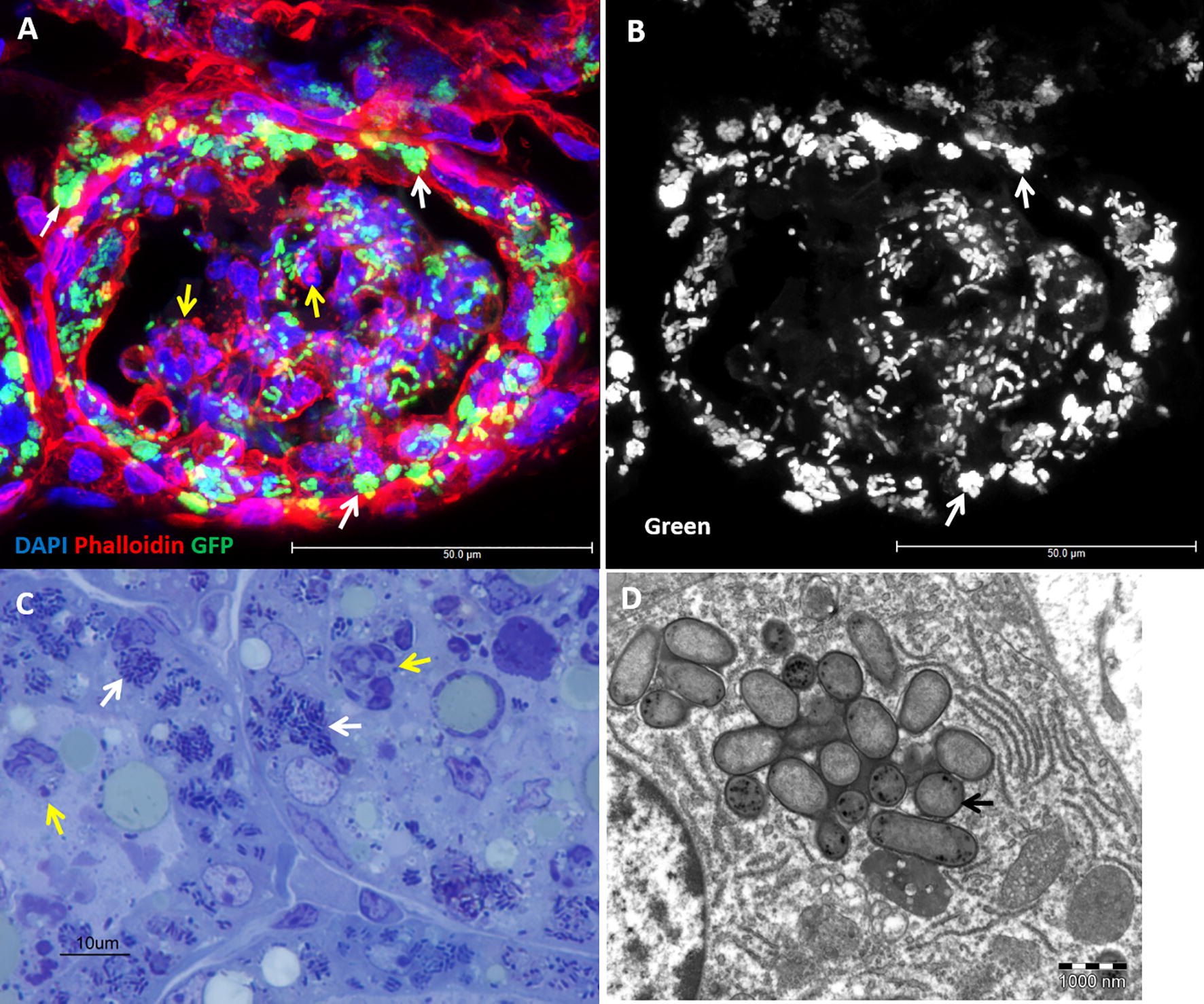
**Mammary pathogenic** ***E. coli*** **(MPEC) form intracellular bacterial communities (IBC) in murine mammary alveolar epithelium.** Lactating C57BL/6 TLR2−/− (**A**, **B**) or C3H/HeJ (**C**, **D**) mice 24 h after challenge by approximately 1000 CFUs via the teat canal. Mammary gland cryosections stained with DAPI (blue) and phalloidin-TRITC (red) (**A**, **B**), thin sections (1 µm) stained with toluidine blue (**C**) and TEM (**D**). Scale bars 50 µm (**A**, **B**), 10 µm (**C**), and 1000 nm (**D**). IBC of GFP-producing bacteria are demonstrated using confocal microscopy (showing a single Z-stack; white arrows in **A**, **B**), toluidine blue staining (white arrows in **C**) and TEM (black arrow in **D**). Inflammation is characterized by massive neutrophil recruitment into the alveolar spaces (yellow arrows in **A**, **C**) interacting with free and phagocytosed bacteria (GFP in **A**, **B** and toluidine blue in **C**). All images are representative of the entire sample. The histological morphology and pathology results were very similar for each gland in a given mouse and between mice.

**Figure 2:**
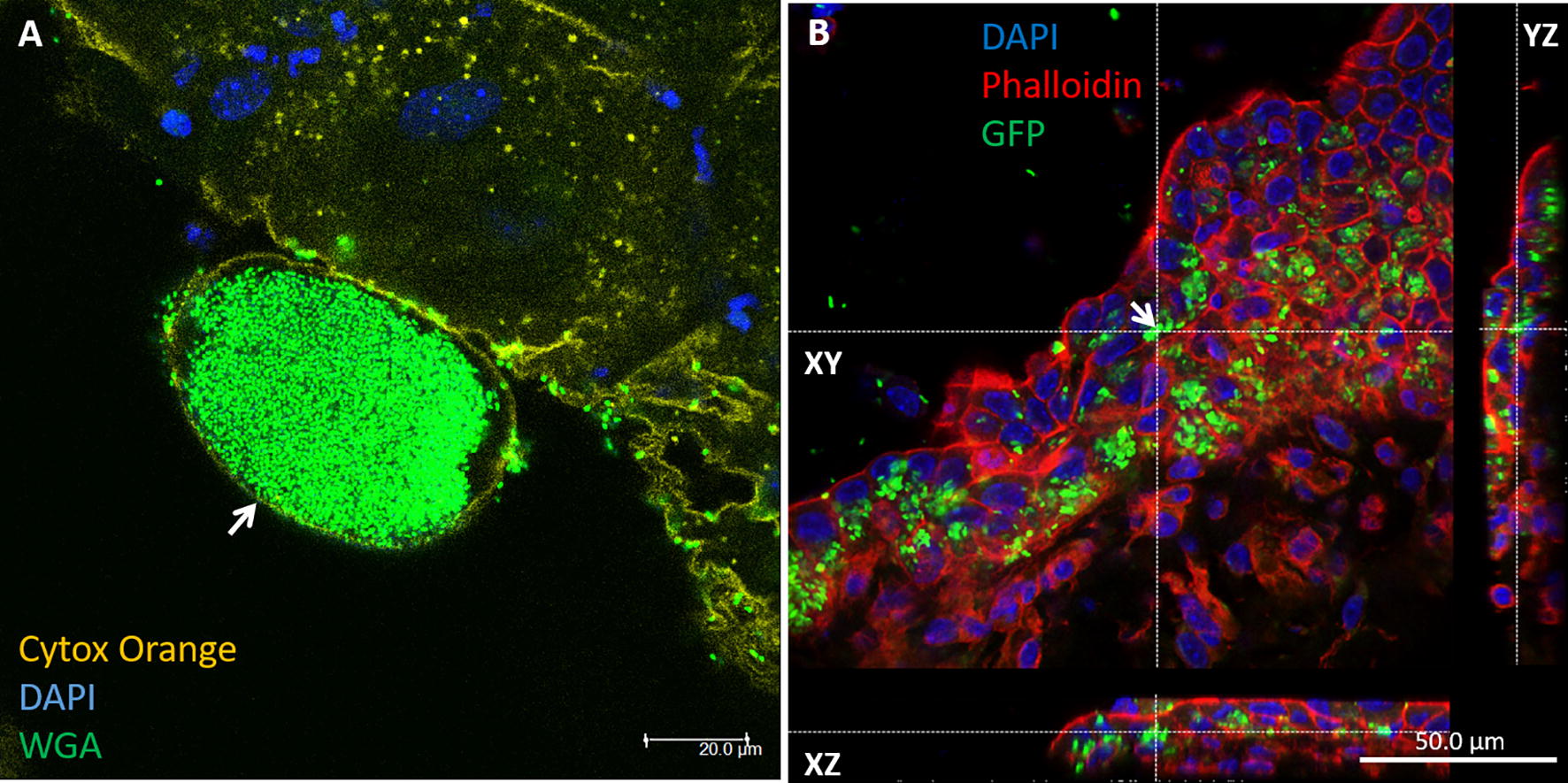
**Intracellular bacterial communities (IBC) in urinary bladder transitional epithelial cell and gall bladder mucosal epithelium.** Female C57BL/6 mice were challenged by intra-urethral inoculation with 10^7^ CFUs human urinary pathogenic *E. coli* strain UTI89 and its bladder was harvested 24 h after infection. Whole mounts of urinary bladder were stained with cytox orange (light green in **A**), DAPI (blue in **A**) and WGA (false colored dark green in **A**). A single Z-section obtained by confocal laser microscopy demonstrates a large aggregation of intracellular bacterial community (IBC) in superficial umbrella bladder epithelial cell (arrow in **A**). Female C57BL/6 mice were challenged by injection of 10^5^ CFUs of *Salmonella enterica* serovar Typhimurium/P*oxyS*-*gfp* strain SL1344 into the gall bladder which was harvested 24 h after infection. Whole mounts of gall bladder were stained with DAPI (blue in **B**) and phalloidin-TRITC (red in **B**). A single Z-section obtained by confocal laser microscopy demonstrates IBC of GFP-expressing bacteria in gall bladder epithelial cells. Scale bars 20 µm (**A**) and 50 µm (**B**). All images are representative of the entire sample. The histological morphology and pathology results were very similar for each organ in a given mouse and between mice.

We have previously described, and show here (Figure 1), the formation of IBC in mammary alveolar epithelial cells following teat canal ascending infection with mammary pathogenic *E. coli* (MPEC) in lactating mice [7, 8, 14]. IBC of urinary pathogenic *E. coli* (UPEC) in bladder epithelial cells were also described in mouse UTI model and naturally infected women [15, 16], and are also shown here following trans-urethral ascending infection (Figure 2). Previous studies demonstrated similar communities of *Salmonella enterica* serovar Typhi (S. Typhi) in gall bladder epithelium following systemic or oral challenge in mice [17, 18]. Using a novel model of gall bladder ascending infection in mice, we show here the formation of IBC of S. Typhi in gall bladder epithelial cells (Figure 2).

The above described IBCs were suggested to play important role in the pathogenesis of these disease condition. Moreover, IBCs might provide a potential mechanism of chronic carriage, relapsing disease and bacterial dissemination.

### Bacterial communities are associated with intracellular neutrophils

**Figure 3:**
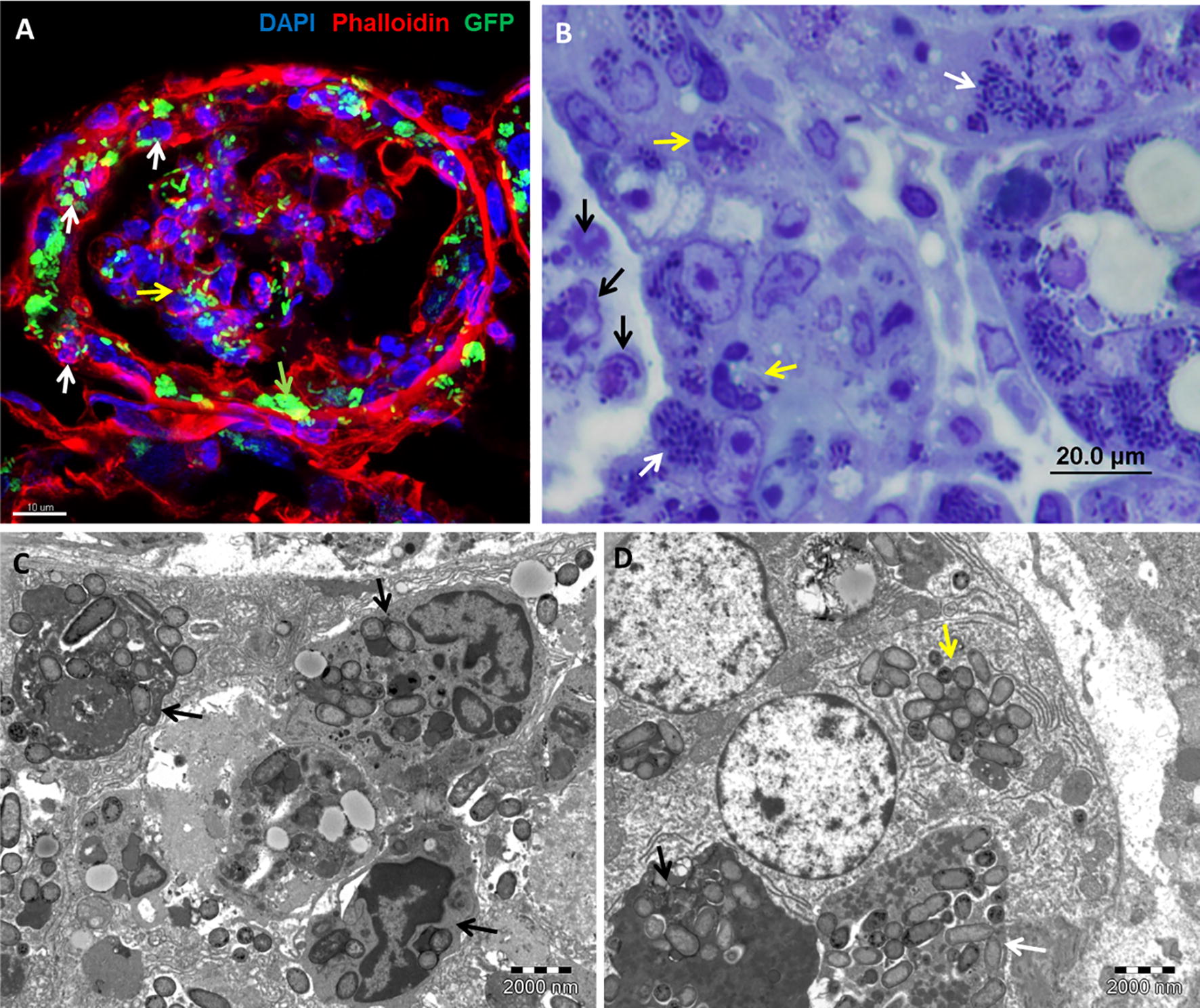
**Bacterial communities are associated with intracellular neutrophils in mammary epithelial cells.** Lactating C57BL/6 TLR2−/− (**A**) or C3H/HeJ (**B**–**D**) mice were challenge by approximately 1000 cfu via the teat canal. Mammary gland cryosections stained with DAPI (blue) and phalloidin-TRITC (red) (**A**), thin sections (1 µm) stained with toluidine blue (**B**) and TEM (**C**, **D**). Scale bars 10 µm (**A**), 20 µm (**B**), and 2000 nm (**C**, **D**). Intracellular neutrophils (white arrows in A) are demonstrated using confocal microscopy (showing a single Z-stack; see also Additional file 1), toluidine blue staining (yellow arrows in **B**) and TEM (black arrows in **C**) and many of these neutrophils are infected with bacteria. Inflammation is characterized by massive neutrophil recruitment into the alveolar spaces (yellow arrows in **A** and black arrows in **B**) interacting with free and phagocytosed bacteria. IBC of GFP-producing bacteria are visible in the alveolar epithelial cells (green arrow in **A** and white arrows in **B**). Free cytosolic bacteria (yellow arrow in **D**) are visible in comparison with bacteria still associated with remnants of hosting neutrophils (black and white arrows in **D**). All images are representative of the entire sample. The histological morphology and pathology results were very similar for each gland in a given mouse and between mice.

**Figure 4:**
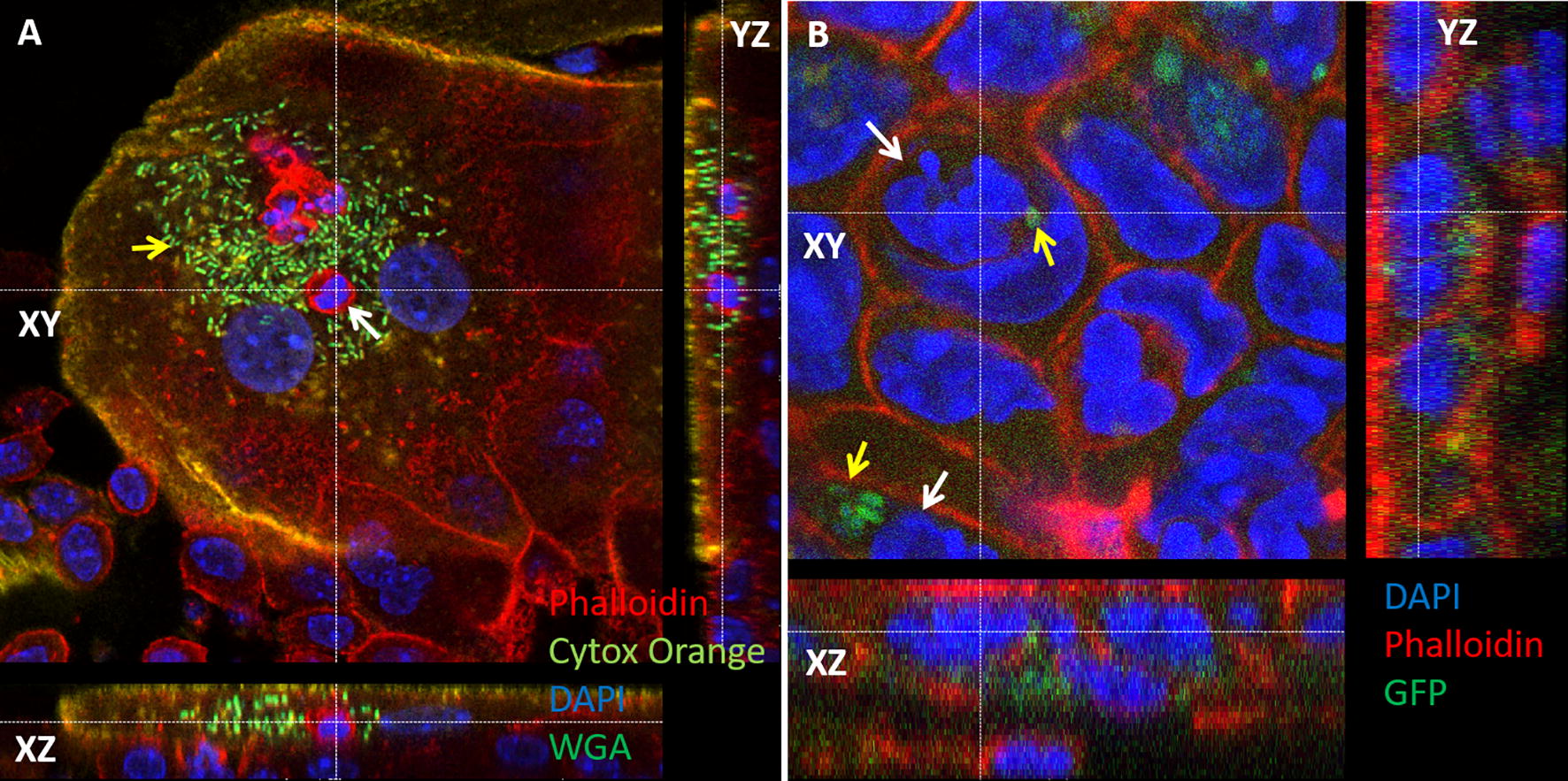
**Bacterial communities are associated with intracellular neutrophils in urinary bladder transitional epithelial cells (A) and gall bladder mucosal epithelial cells (B).** Female C57BL/6 mice were challenged by intra-urethral inoculation with 10^7^ CFUs human urinary pathogenic *E. coli* strain UTI89 and its bladder was harvested 24 h after infection. Whole mounts of urinary bladder were stained with cytox orange (light green in **A**), DAPI (blue in **A**) and WGA (false colored dark green in **A**). Confocal laser microscopy demonstrates a large aggregation of intracellular bacterial community (IBC) in superficial umbrella bladder epithelial cell (yellow arrow in **A**) and intraepithelial neutrophil (white arrow in **A**; see also Additional file 2). Female C57BL/6 mice were challenged by injection of 10^5^ CFUs of *Salmonella enterica* serovar Typhimurium/P*oxyS*-*gfp* strain SL1344 into the gall bladder which was harvested 24 h after infection. Whole mounts of gall bladder were stained with DAPI (blue in **B**) and phalloidin-TRITC (red in **B**). Confocal laser microscopy demonstrates GFP-expressing bacteria in gall bladder epithelial cells and intraepithelial neutrophils (white arrows in **B**; see also Additional file 3). The *xy* images are on the plan indicated by the horizontal and vertical dashed lines shown in the *xz* and *yz* images, respectively. Original magnification X63 (**A**, **B**). All images are representative of the entire sample. The histological morphology and pathology results were very similar for each organ in a given mouse and between mice.

In tracking the mechanism of MPEC invasion into mammary epithelial cells, we noted the presence of infected neutrophils and bacterial communities in the cytoplasm of these cells (Figure 3, Additional file 1). Using the mouse UTI model and gall bladder ascending infection model, we also observed the co-existence of neutrophils and bacterial communities of *E. coli* (Figure 4A, Additional file 2) and S. Typhi (Figure 4B, Additional file 3), respectively, in the cytoplasm of bladder epithelial cells.

### Entry of neutrophils into mammary epithelial cells

**Figure 5:**
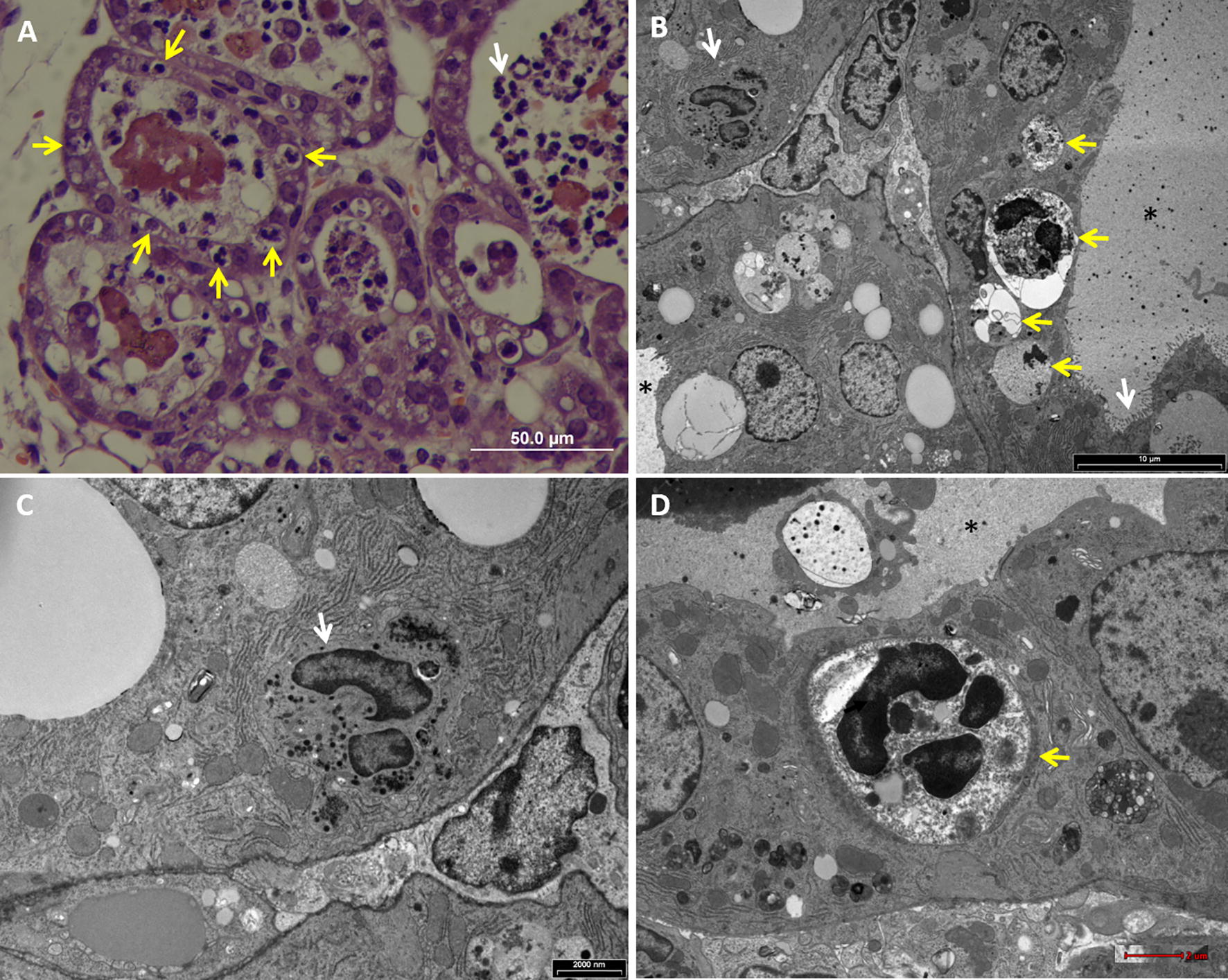
**Live and apoptotic intraepithelial neutrophils in mammary epithelial cells.** Lactating C57BL/6 mice where infused with 10^6^ CFUs of viable *E. coli* DH5α bacteria. H&E staining of paraffin sections (**A**) and transmission electron microscopy imaging (**B**–**D**) of mammary tissues 24 h after challenge. Mastitis is characterized by massive recruitment of neutrophils into the alveolar spaces (white arrow in **A**; see also Additional file 4B) and alveolar epithelial cells containing intraepithelial neutrophils (yellow arrows in **A**). Morphologically normal (white arrow in **B**, **C**) and apoptotic neutrophils (yellow arrows in **B**, **D**) are visible in mammary alveolar epithelial cells. Microvilli are visible on the apical membrane of the mammary epithelial cells (white arrow in **B**) and milk space is marked by asterisk in **B**. Scale bars 50 µm (**A**), 10 µm (**B**), 2000 nm (**C**) and 2 µm (**D**). All images are representative of the entire sample. The histological morphology and pathology results were very similar for each gland in a given mouse and between mice.

To better describe the process of neutrophil entry we have used two murine mastitis model systems; (1) MPEC challenge in C57BL6 TLR2-deficient mice where both IBCs and intraepithelial neutrophils were observed (Figures 1, 3A, Additional file 1), and (2) a novel model where we infected the mammary gland of C57BL/6 lactating mice with the non-pathogenic laboratory strain *E. coli* DH5α, which is unable to replicate in the mammary gland (Additional file 4A) or to survive in neutrophils. Although this strain failed to replicate in the mammary gland following IMM challenge with 10^6^ CFUs, this microbial biomass was sufficient to elicit neutrophil recruitment into the alveolar spaces (Additional file 4B). Interestingly, while entry of neutrophils could be observed in many alveolar epithelial cells (> 1 intraepithelial neutrophil in >50% of inflamed alveoli), intracellular replication and IBC formation was not observed with this strain (Figure 5).

Transmission electron microscopy (TEM) of mammary tissues obtained from the two mastitis models revealed the presence of two intracellular neutrophil populations; (1) viable and active (Figures 5B and C, Additional files 5A, 6), and (2) apoptotic (Figures 5B and D, Additional file 7). Viable (or live) and active neutrophils were highly polymorphic with normal polymorphonuclear nucleus, normal cytoplasmic granules and intact cell membrane. Many of these cells were associated with a double membrane compartment tethered to the junctional complexes of the adjacent epithelial cells (Additional files 5, 6). In contrast, early and late apoptotic changes were visible in many intracellular neutrophils (Figure 5, Additional file 7). Early apoptotic changes included condensed nuclear chromatin, rounded nuclear profiles, glycogen granules depletion, cytoplasmic vacuoles, and preservation of cytoplasmic granules. Late changes included small and highly condensed nucleus (apoptotic body), extensive cytoplasmic vacuolization, loss of granules and complete loss of membranous compartments.

These observation lead us to hypothesize that upon contact with the apical membrane and junctional complexes of polar barrier epithelial cells, recruited neutrophils reverse and crawl into a double membrane compartment created by the adjacent host cells membrane (Additional files 7B, 8). These membrane compartments represent an intermediate step in the entry process, at which scission from the plasma membrane has not been completed although the entrapped neutrophils are segregated and protected from the extracellular milieu. Considering the short life span of activated and infected neutrophils, these intraepithelial viable neutrophils become apoptotic, lose their membranous compartment and release their cargo into the cytoplasm of the host cells.

### Entry of neutrophils into mammary and urinary epithelial cell lines

**Figure 6:**
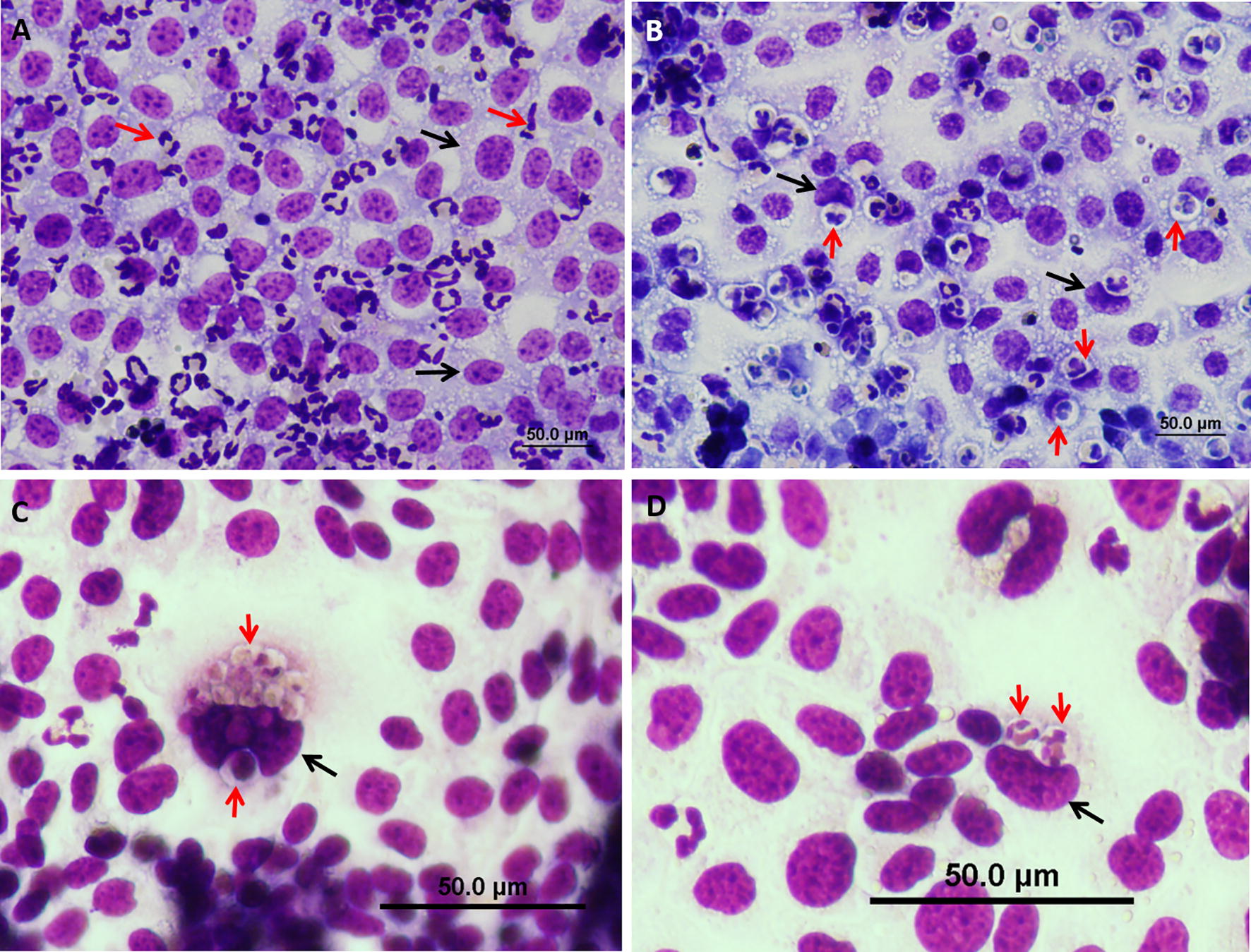
**In vitro entry of neutrophils into mammary epithelial cells.** Isolated fresh bovine blood neutrophils were placed on a monolayer of EPH-4 cells grown on glass cover slides in 24 wells culture plate. After 12 h cells were fixed with PFA and stained with Diff Quick and examined by bright light microscopy. Neutrophils (red arrows in **A**) adhere to EPH-4 cells (black arrows in **A**) before entry. Intraepithelial neutrophils (red arrows in **B**–**D**) are visible in a clear cytoplasmic vacuole indenting the nuclei of the host epithelial cell (black arrows in **B**–**D**). Some epithelial cells contain large numbers of intraepithelial neutrophils (**C**) while most contain one or two intraepithelial neutrophils (**D**). Scale bars 50 µm (**A**, **B**), and 50 µm (**C**, **D**). All images are representative of > 3 similar experiments.

**Figure 7:**
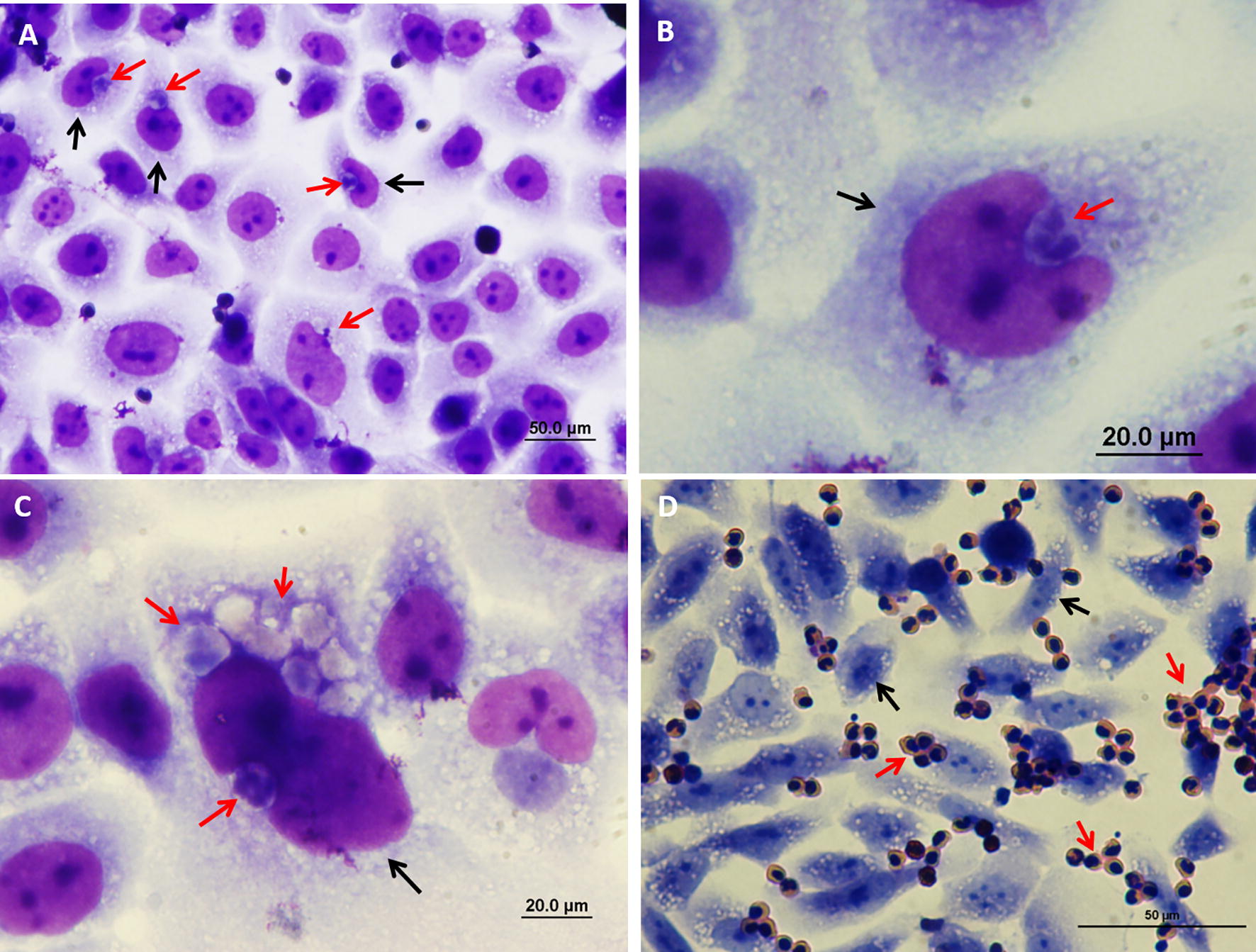
**In vitro entry of neutrophils into human bladder epithelial cells.** Isolated fresh bovine blood neutrophils were placed on a monolayer of human epithelial cell line 5637 cells (black arrows in **A**–**D**) grown on glass cover slides in 24 wells culture plate. After 12 h cells were fixed with PFA and stained with Diff Quick and examined by bright light microscopy. Intraepithelial neutrophils (red arrows in **A**–**C**) are visible in bladder cells (black arrows in **A**–**C**). Intraepithelial neutrophils are visible in a clear cytoplasmic vacuole displacing the epithelial nuclei. Some epithelial cells contain large numbers of intraepithelial neutrophils (**C**) while most contain one or two intraepithelial neutrophils Intracellular neutrophils (red arrow in **B**). PFA-fixed neutrophils fail to internalize and are visible as aggregates of round cells adhered to the epithelial cells (red arrows in **D**). Scale bars 50 µm (**A**, **D**) and 20 µm (**B**, **C**). All images are representative of > 3 similar experiments.

**Figure 8:**
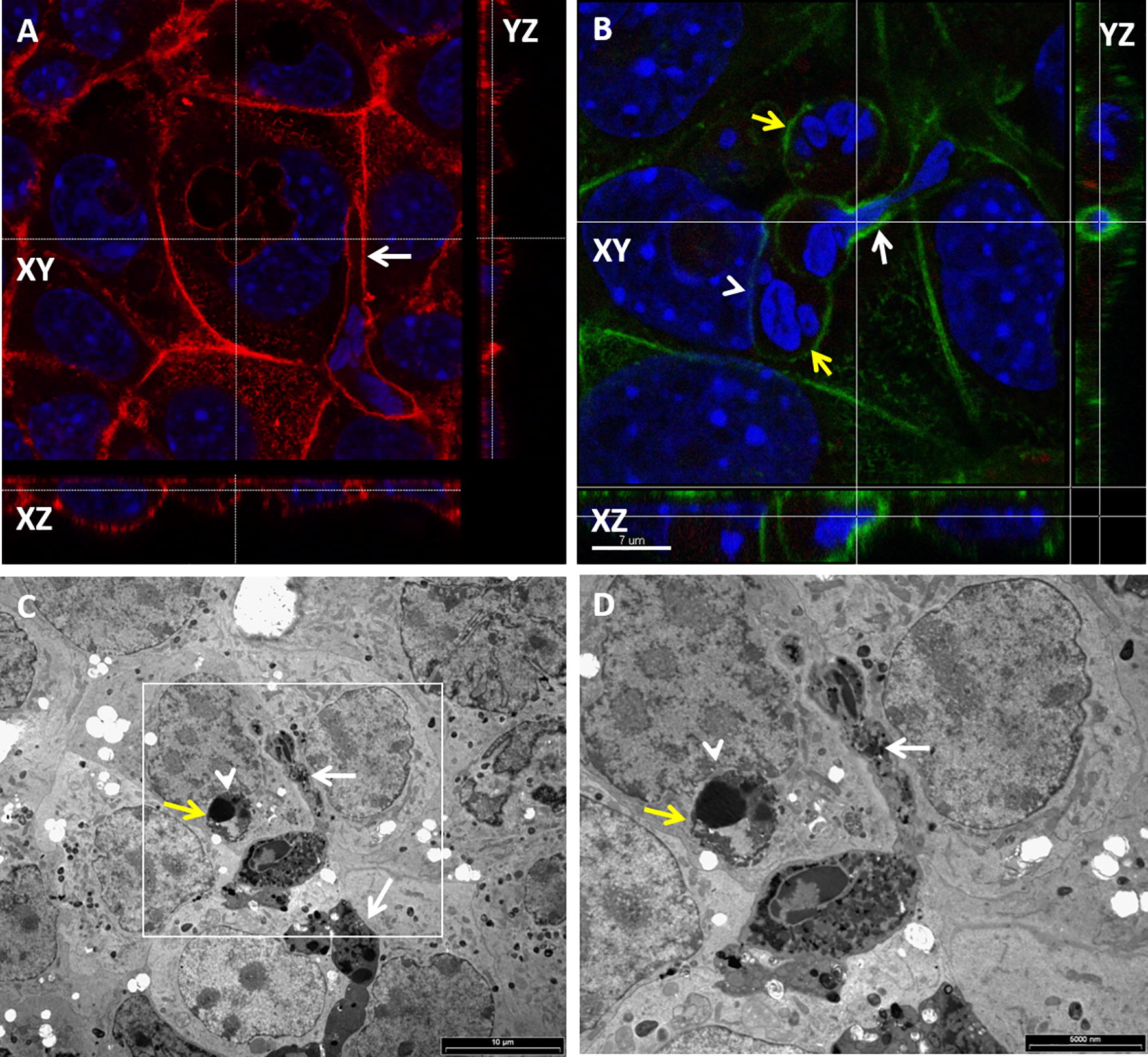
**Entry of mammary and urinary epithelial cells by neutrophils.** Isolated fresh bovine blood neutrophils were placed on a monolayer of murine mammary EPH-4 cells (**A**, **B**) or human urinary epithelial cell line 5637 (**C**, **D**) grown on glass cover slides in 24 wells culture plate. After 12 h cells were fixed with PFA and stained with DAPI (blue in **A**, **B**) and phalloidin-TRITC (red in **A**, **B**) and or prepared for TEM (**C**, **D**). Representative images of confocal laser microscopy (**A**, **B**) and TEM (**C**, **D**). The *xy* image is on the plan indicated by the horizontal and vertical dashed lines shown in the *xz* and *yz* images, respectively (**A**, **B**). Scale bars 7 µm (**A**, **B**), 10 µm (**C**) and 5000 nm (**D**). Entry preceded by elongation and crawling of live neutrophils along the intercellular borders of the polar epithelial cell monolayer (white arrows in **A**, **C**, **D**). The entry process is associated with the formation of an actin-rich tunnel (white arrow in **B**) and required extensive elongation and rearrangement of the nucleus to accommodate this entrance route. Intraepithelial live (yellow arrows in **B**) and apoptotic (yellow arrows in **C**, **D**) neutrophils are also visible. All images are representative of > 3 similar experiments.

**Figure 9:**
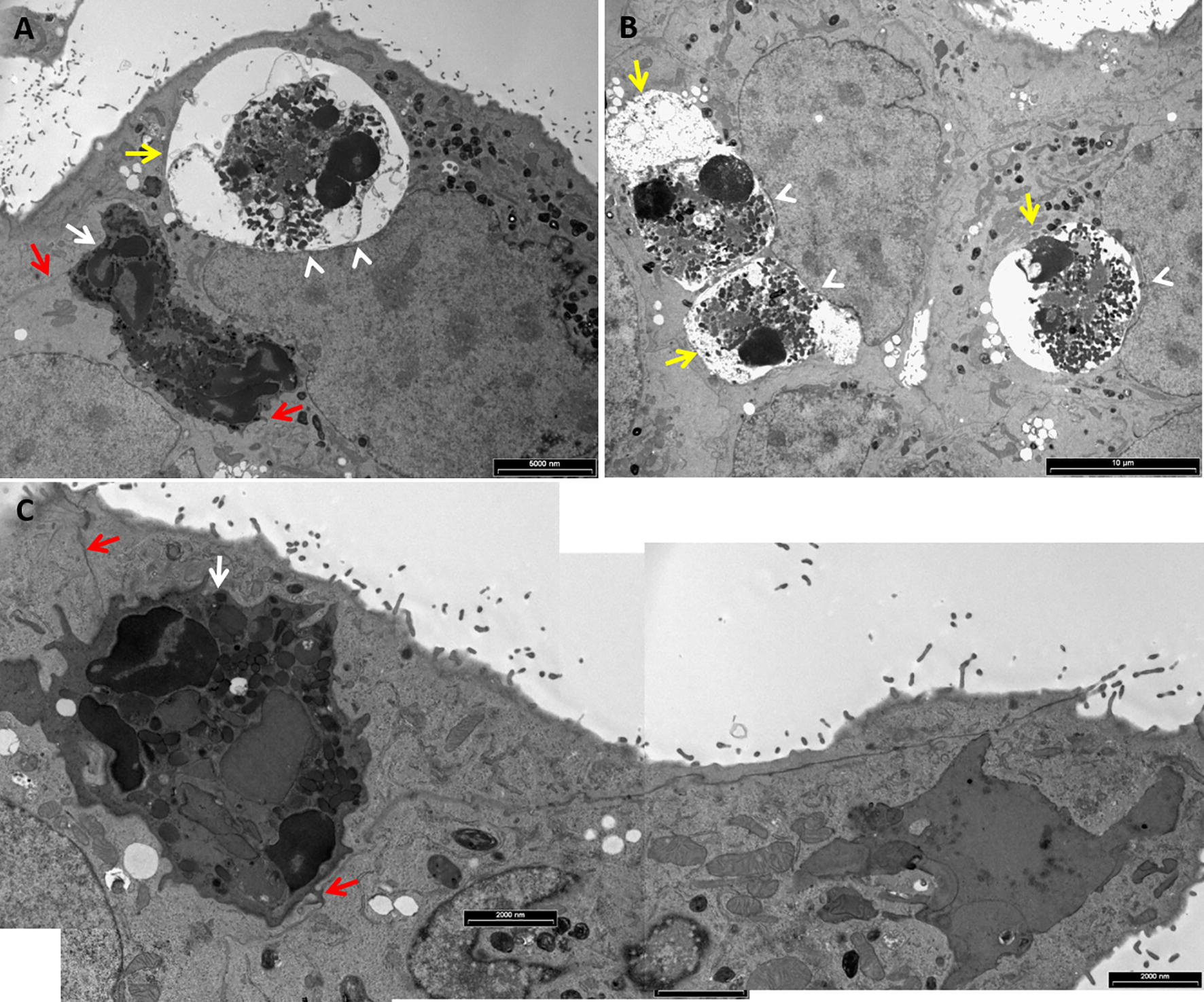
**Live and apoptotic neutrophils in human urinary epithelial cells.** Isolated fresh bovine blood neutrophils were placed on a monolayer of human urinary epithelial cell line 5637 grown on glass cover slides in 24 wells culture plate. After 12 h cells were fixed with PFA and imaged using TEM. Scale bars 6000 nm (**A**), 10 µm (**B**) and 2000 nm (**C**). Live neutrophils (white arrows in **A**, **C**) are enclosed in a double membrane compartment tethered to host cells membranes and junctional complex (red arrows in **A**, **C** and Additional file 9). Live and apoptotic neutrophils are clearly identified by their multilobulated nuclei the presence of typical glycogen granules in their cytoplasm. Apoptotic neutrophils are visible as a clear vacuole and typically displacing the nuclei of host epithelial cells (white arrow heads in **A**, **B**). All images are representative of > 3 similar experiments.

To further the observation of neutrophil entry we used an in vitro cell system of polar murine mammary (EPH-4) and human bladder (5637) epithelial cell line which were layered on the apical side with normal bovine blood neutrophils. Numerous intraepithelial neutrophils were observed in monolayers of the two epithelial cell lines (Figures 6, 7). Neutrophils were unable to enter sparsely grown or detached epithelial cells. Neutrophil entry preceded by elongation of the normally round blood neutrophils and crawling along the intercellular borders of the polar epithelial cell monolayer (Figure 8). The entry process was associated with the formation of an actin-rich tunnel which required extensive elongation and rearrangement of the nucleus to accommodate this entrance route (Figure 8B). Intraepithelial neutrophils were not observed following layering of the epithelial monolayer with normal, formalin-fixed, “mummified” or apoptotic neutrophils (Figure 7D, Additional file 9), thus further underlying that neutrophil entry does not represent phagocytosis of apoptotic cells. TEM analysis of intraepithelial neutrophils in monolayers revealed the presence of viable and apoptotic neutrophil in the same epithelial cell (Figure 9). Viable neutrophils were also enclosed in a double membrane compartment tethered to junctional complexes of host epithelial cells thus clearly recapitulating our in vivo observations (Figure 9, Additional file 10).

**Figure 10:**
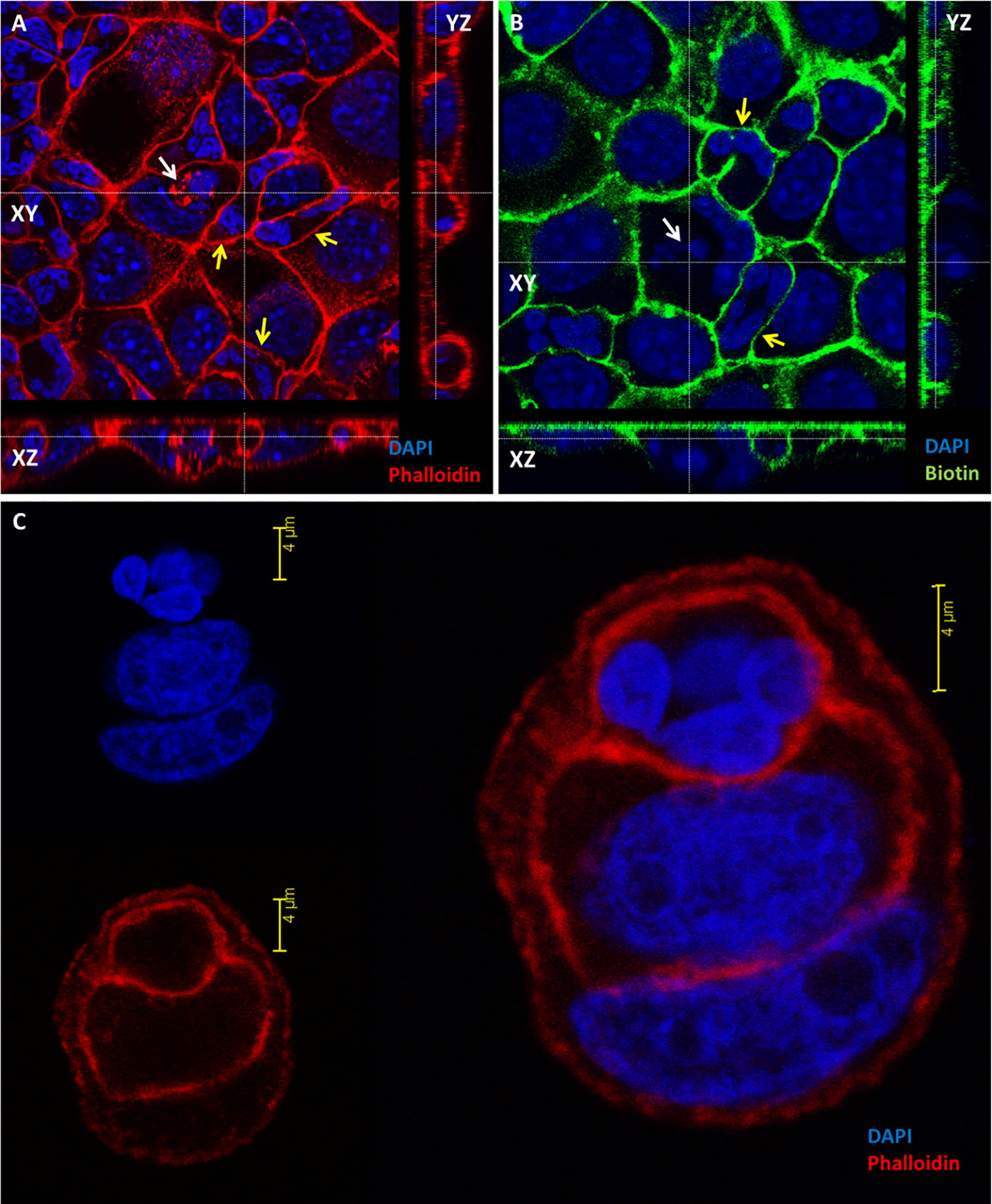
**Complete entry of neutrophils in murine mammary epithelial cell line EPH4 (A, B) and human bladder epithelial cell line 5637.** Isolated fresh bovine blood neutrophils were placed on a monolayer of EPH4 cells or human bladder epithelium cell line 5637 grown on glass cover slides in 24 wells culture plate. To ascertain complete entry of neutrophils into the EPH-4 cells and loss of membrane invagination, the epithelial monolayer with intraepithelial neutrophils (white arrow in **A**) were assayed for protection from surface biotinylation (white arrow in **B**). PFA-fixed monolayers were stained with phalloidin (red in **A**) and DAPI (blue in **A**, **B**) for epifluorescence and confocal microscopy. The *xy* image is on the plan indicated by the horizontal and vertical dashed lines shown in the *xz* and *yz* images, respectively. Intraepithelial neutrophils were protected from surface labeling with biotin (white arrow in **B**), indicating their complete entry while crawling and invaginating neutrophils (yellow arrows in **A**, **B**) are labeled. **A**, **B** Original magnification ×63. To further examine intraepithelial neutrophils in human bladder epithelium cell line 5637, single cell suspension of trypsinized monolayer with intraepithelial neutrophils was cytospun onto glass slides, fixed with 2% PFA and stained with DAPI and phalloidin (**B**). Analysis by confocal microscopy demonstrated complete entry of neutrophils within epithelial cells or, in some cases, one neutrophil within an epithelial cell that was within a third epithelial cell (**C**). Scale bar 4 µm (**C**). All images are representative of > 3 similar experiments.

To demonstrate the scission of invaginated epithelial membranes from the entry point, EPH-4 epithelial monolayers with intracellular neutrophils were assayed for protection from surface biotinylation. While elongated neutrophils crawling along the epithelial cell borders were clearly labeled, intraepithelial neutrophils were protected from surface labeling with biotin, indicating their complete entry rather than invagination of epithelial membrane (Figures 10A and B). To further demonstrate complete neutrophils entry into human bladder epithelium cell line 5637, monolayer was trypsinized and washed to a single cell suspension and cytospun onto glass slides. Following fixation and fluorescence staining, analysis by confocal microscopy demonstrated complete entry of neutrophils into epithelial cells or, in some cases, one neutrophil within an epithelial cell that was within a third epithelial cell (Figure 10C). This unusual phenomenon, which has not been observed in monolayers, most probably represent entosis of epithelial cell hosting a neutrophil into another cell which is known to occur in single cell suspensions of detached epithelial cells [23].

## Discussion

We show here the presence of live and apoptotic neutrophils in the cytoplasm of inflamed mammary, urinary and gall bladder epithelial cells. The entry process commences with adherence of transmigrated neutrophils to the apical membrane of inflamed epithelial cells. Next, nuclear rearrangement and elongation was associated with extensive actin polymerization that enabled neutrophils to crawl and invaginate the apical membrane into cytoplasmic double membrane compartments. Scission of the invaginated cell membrane from the entry point was demonstrated by surface biotinylation. Furthermore, using TEM we demonstrated loss of the membranous compartment surrounding apoptotic neutrophils in the epithelial cell cytoplasm.

Uptake of apoptotic cells, including neutrophils, by professional and non-professional phagocytes (such as barrier epithelial cells) is a major inflammatory regulation mechanism [11, 24]. The presence of apoptotic cells and bodies within cells, including epithelial cells, decrease pro-inflammatory and promote anti-inflammatory response by host cells [25–27]. Specifically, neutrophil apoptosis and efferocytosis are integral modulatory mechanisms that constrain inflammation and contribute to its successful resolution. Furthermore, intracellular release of neutrophilic cell content might also contribute to resolution of inflammation. We show here that although epithelial cells are able to uptake apoptotic neutrophils, live neutrophils are also able to actively invade epithelial cells and meet their inevitable demise in the safe haven of a double membrane intracellular refuge.

Based on current knowledge we can only speculate that this incredible phenomenon contributes to the resolution of inflammation at the investigated sites. An interesting extrapolation from this work is that enhancing neutrophil invasion might contribute to inflammatory resolution.

Collectively, the data presented in this work provide several key insights. First, uptake of apoptotic neutrophils by barrier epithelial cells is most probably rare. However, apoptosis is a spectrum of highly dynamic process and we cannot exclude the possibility that early apoptotic neutrophils might still maintain their capacity to enter the epithelial cells or that the expression of early apoptotic markers is even essential for the entry process. Second, neutrophils are inherently pre-programed to die by constitutive apoptosis, thus, apoptotic neutrophils in epithelial cells are the inevitable descents of live or early apoptotic neutrophils that actively invaded these cells. Neutrophil entry is an active process requiring close contact and crosstalk between neutrophils and polar epithelial cells. This step was followed by deformation and elongation of the nucleus and, once entry was completed, the nucleus was refolded into a roundish, multilobular shape. This process was associated with extensive actin polymerization around the elongating nucleus as previously described for transmigrating neutrophils [28, 29]. We further speculate that the mechanism of neutrophil entry to mammary and urinary epithelium is distinct from phagocytosis of apoptotic cells and achieved by active invasion of normal neutrophils. Moreover, apoptosis is associated with chromatin condensation, increased rigidity and reduced deformability, all of which are expected to perturb the agile maneuvering exhibited here. The entry process is not species-specific and was demonstrated using various combinations of bovine, murine and human neutrophils and epithelial cells (our unpublished data). Although the molecular mechanism underlying entry is currently unknown, this cross-species compliance indicate a highly conserved and advantageous process. Molecular mechanisms involved in reverse migration of neutrophils might be involved in the first stage of this process [3, 4]. While overlooking neutrophil entry, we have previously demonstrated that adherence of neutrophils to the apical membrane of polar mammary epithelium was dependent on CD44 [30], which is known to play a role in neutrophil epithelial transmigration and efferocytosis [31, 32]. CD44 is also expressed on urinary and gall bladder epithelium and might similarly contribute to neutrophil adhesion and entry into these epithelial cells [33, 34]. Both CD44 and intercellular adhesion molecule 1 (ICAM-1) are expressed on the apical membrane of epithelial cells lining mucosal structures and were implicated to play a role in luminal interaction with recruited neutrophils [10]. Moreover, inflammatory mediators increase the expression of ICAM-1 in mammary gland, urinary and gall bladder epithelial cells [35–37] which together with CD44 might be part of the entry mechanism of neutrophils into these cells.

Third, the presence of infected neutrophils in epithelial cells suggests that this route is used by some pathogens to invade and establish bacterial communities in barrier epithelial cells. IBC were previously reported in mammary [8, 14], urinary [15] and gall bladder epithelial cells [18]. The mechanism of IBC formation in these tissues is currently unknown and survival in recruited neutrophils actively invading the epithelial cell layers of these organs might have been overlooked as a possible mechanism. Mammary and urinary IBC were successfully established in mastitis and cystitis mouse models. However, while the clinical relevance of IBCs is well established in human UTI [16, 38], their clinical relevance in persistent *E. coli* mastitis is not clear [39, 40]. Chronic asymptomatic carriage and fecal shedding of Salmonella organisms are known to occur in diverse species such as humans and cattle and constitute a major public health concern [17, 18]. The gallbladder is considered the main reservoir and a source of intermittent release of bacteria into the gut leading to shedding and spread of contaminated excreta. Although Salmonella biofilm growing on gallstones is one possible mechanism of chronic carriage, chronic carriage still known to occur in the absence of gallstones, especially in farm animals. Salmonella IBC formation in gallbladder epithelium is a possible mechanism, however, the absence of small animal model system renders exploration of this mechanism difficult. Using a novel model of ascending cholecystitis we show here that luminal infection of the murine gallbladder with Salmonella Typhi resulted in acute inflammation characterized by massive neutrophil recruitment. Moreover, infected neutrophils actively invaded the gallbladder epithelium and Salmonella IBC were visible. This model system enable further research into the mechanisms of Salmonella carriage in the gallbladder. Chronic Salmonella Typhi infection was also associated with increased risk for gallbladder cancer (GBC) in human patients [41]. Our newly developed model system might also be instrumental in the study of the mechanism underlying this association.

## Additional files

**Additional file 1.** **Fluorescence channels comprising Figure** **3****A.** Mammary gland cryosections stained with DAPI (blue) and phalloidin-TRITC (red) and GFP-expressing MPEC bacteria (**A**). Confocal microscopy showing a single Z-stack of merged image (**A**) and separate channels (**B**–**D**) in black and white. Scale bars 10 µm.

**Additional file 2.** **Bacterial communities are associated with intracellular neutrophils in urinary bladder transitional epithelial cells.** Female C57BL/6 mice were challenged by intra-urethral inoculation with 10^7^ CFUs human urinary pathogenic *E. coli* strain UTI89 and its bladder was harvested 24 h after infection. Whole mounts of urinary bladder were stained with phalloidin-TRITC (**A**), cytox orange (**B**), DAPI (**C**). Confocal laser microscopy demonstrates a large aggregation of intracellular bacterial community (IBC) in superficial umbrella bladder epithelial cell (yellow arrows in **A**, **B**) and intraepithelial neutrophil (white arrows in **A** and **C**). Composite image is presented in **D**. The *xy* image is on the plan indicated by the horizontal and vertical dashed lines shown in the *xz* and *yz* images, respectively. Scale bars 20 µm (**A**–**D**).

**Additional file 3.** **Bacterial communities are associated with intracellular neutrophils in gall bladder mucosal epithelial cells.** Fluorescence channels comprising Figure 4B. Female C57BL/6 mice were challenged by injection of 10^5^ CFUs of *Salmonella enterica* serovar Typhimurium/P*oxyS-gfp* strain SL1344 into the gall bladder which was harvested 24 hours after infection. Whole mounts of gall bladder were stained with DAPI (**C**, **D**) and phalloidin-TRITC (**B** and **D**). Confocal laser microscopy demonstrates GFP-expressing bacteria in gall bladder epithelial cells (yellow arrows in **A** and **C**, **D**) and intraepithelial neutrophils (white arrows in **B**–**D**). Composite image is presented in **D**. Original magnification X63 (**A**, **B**).

**Additional file 4.** ***E. coli*** **DH5α is not mammary pathogenic and does not replicate in the mammary gland following intramammary challenge. A**, Mammary bacterial burden 24 hours after intramammary challenge with 10^1^ to 10^6^ viable *E. coli* DH5α into wild-type C57BL/6 mice are shown. Each symbol represents one gland and all bars represent the median. Medians CFU/gr were analyzed using One-Sample Wilcoxon Signed Rank Test and the null hypothesis was median of CFUs/gr equals to the challenge dose (10^1^ to 10^6^), none of which were statistically significant. H&E staining of formalin-fixed mammary tissues (**B**–**D**). Mammary gland with massive recruitment of neutrophils into the alveoli (white arrows in **B**) 24 h after infusion with 10^6^ viable *E. coli* DH5α. This is better seen in Figure 5A (white arrow), which is an enlargement of the boxed area in **C**. Scale bar 200 µm (**B**).

**Additional file 5.** **Live neutrophil (white arrow in A) in mammary epithelial cell enclosed in a double membrane compartment tethered to epithelial cell junctional complex.** Lactating C57BL/6 mice where infused with 10^6^ CFUs of viable *E. coli* DH5α bacteria. Transmission electron microscopy imaging of mammary tissues 24 hours after challenge. Boxed areas in **A** is enlarged in **B** and boxed areas in **B** are enlarged in **C**, **D**). Tethering of neutrophil to epithelial junctional complex is visible (black arrows in **B**, **D**). Microvilli and milk space (black asterisk * in **C**) are visible adjacent to the epithelial cell junctional complex (Black arrow in **C**). Tethering of double membrane to the basolateral membrane of host epithelial cell is also visible (yellow arrows in **B** and **D**). Scale bars 2000 nm (**A**), 1000 nm (**B**), and 500 nm (**C**, **D**).

**Additional file 6.** **Live neutrophil (white arrow in A) in mammary epithelial cell enclosed in a double membrane compartment tethered to epithelial cell junctional complex** (**black arrows in B).** Lactating C57BL/6 TLR2−/− mice were challenge by approximately 1000 CFUs via the teat canal. Transmission electron microscopy imaging of mammary tissues 24 h after challenge. Boxed area is enlarged in **B**. Scale bars 2000 nm (**A**), and 500 nm (**B**).

**Additional file 7.** **Entry and apoptosis of neutrophils in mammary epithelium.** Lactating C57BL/6 mice where infused with 10^6^ CFUs of viable *E. coli* DH5α bacteria. Transmission electron microscopy imaging of mammary tissues 24 h after challenge. Live neutrophil adhering to the apical membrane of alveolar epithelial cell (black arrow in **A**) and commencing the entry process (black arrow in **B**; see enlarged details in Additional file 8). Early (yellow arrow in **A**) and late (yellow arrow in **B**, **C**) apoptosis of neutrophils are also visible. Scale bars 2 µm (**A**), 10 µm (**B**), and 2000 nm (**C**).

**Additional file 8.** **Commencement of entry by live neutrophil (black arrow in A) interacting with microvilli (black arrows in B-D) on the apical membrane of alveolar epithelial cell.** Lactating C57BL/6 mice where infused with 10^6^ CFUs of viable *E. coli* DH5α bacteria. Transmission electron microscopy imaging of mammary tissues 24 h after challenge. Boxed areas in **B** are enlarged in **C** and **D**. The alveolar milk space in indicated by * in A. Scale bars 2000 nm (**A**, **B**), and 200 nm (**C**, **D**).

**Additional file 9.** **Viability is essential for entry of neutrophils into mammary epithelial cells.** Fresh viable, PFA-fixed and UV-treated apoptotic neutrophils were layered over a monolayer of polar mammary epithelial cell line EPH4 and evaluated microscopically for neutrophil internalization after 24 h of co-culture as described in the materials and methods. Neutrophils were cytospun onto glass slides and stained with Diff Quick for microscopic evaluation (**A**). Neutrophil viability and apoptosis were quantified using FACS analysis following staining with propidium iodide (PI in **B**) and Annexin V FITC (**C**). Mean percentage (± SD) of epithelial cells with internalized cells following co-culture with viable, fixed and UV-treated apoptotic neutrophils is presented in **D**. Results of a representative experiment out of three. Mean % cell-in-cell following co-culture with viable neutrophils was compared by unpaired t test using GraphPad Prism 6 (GraphPad Software, Inc.) and was significantly different from all other groups; *P < 0.005. Scale bars 10 µm (**A**).

**Additional file 10.** **Intracellular live neutrophil is enclosed in double membrane compartment (enlarged details of Figure** **9****A).** Isolated fresh bovine blood neutrophils were placed on a monolayer of human urinary epithelial cell line 5637 grown on glass cover slides in 24 wells culture plate. After 12 h cells were fixed with PFA and imaged using TEM. Scale bars 2000 nm (**A**) and 200 nm (**B**–**D**). Top, middle and bottom boxed areas in **A** are enlarged in **B**, **C** and **D**, respectively. Live neutrophil (white arrow in **A**) is enclosed in a double membrane compartment (red arrows in **A**–**D**). Neutrophil is clearly identified by its multilobulated nucleus and the presence of typical glycogen granules in the cytoplasm (white arrow heads in **C**, **D**).

## Abbreviations

IBC: intracellular bacterial communities
UTI: urinary tract infection
MPEC: mammary pathogenic *E. coli*
UPEC: urinary pathogenic *E. coli*
TEM: transmission electron microscopy
