# Supplementary figures and images for: Intraepithelial neutrophils in mammary, urinary and gall bladder infections
Source: Vet Res. 2019 Jul 19;50:56. doi: 10.1186/s13567-019-0676-5 (PMC6642505; doi:10.1186/s13567-019-0676-5)

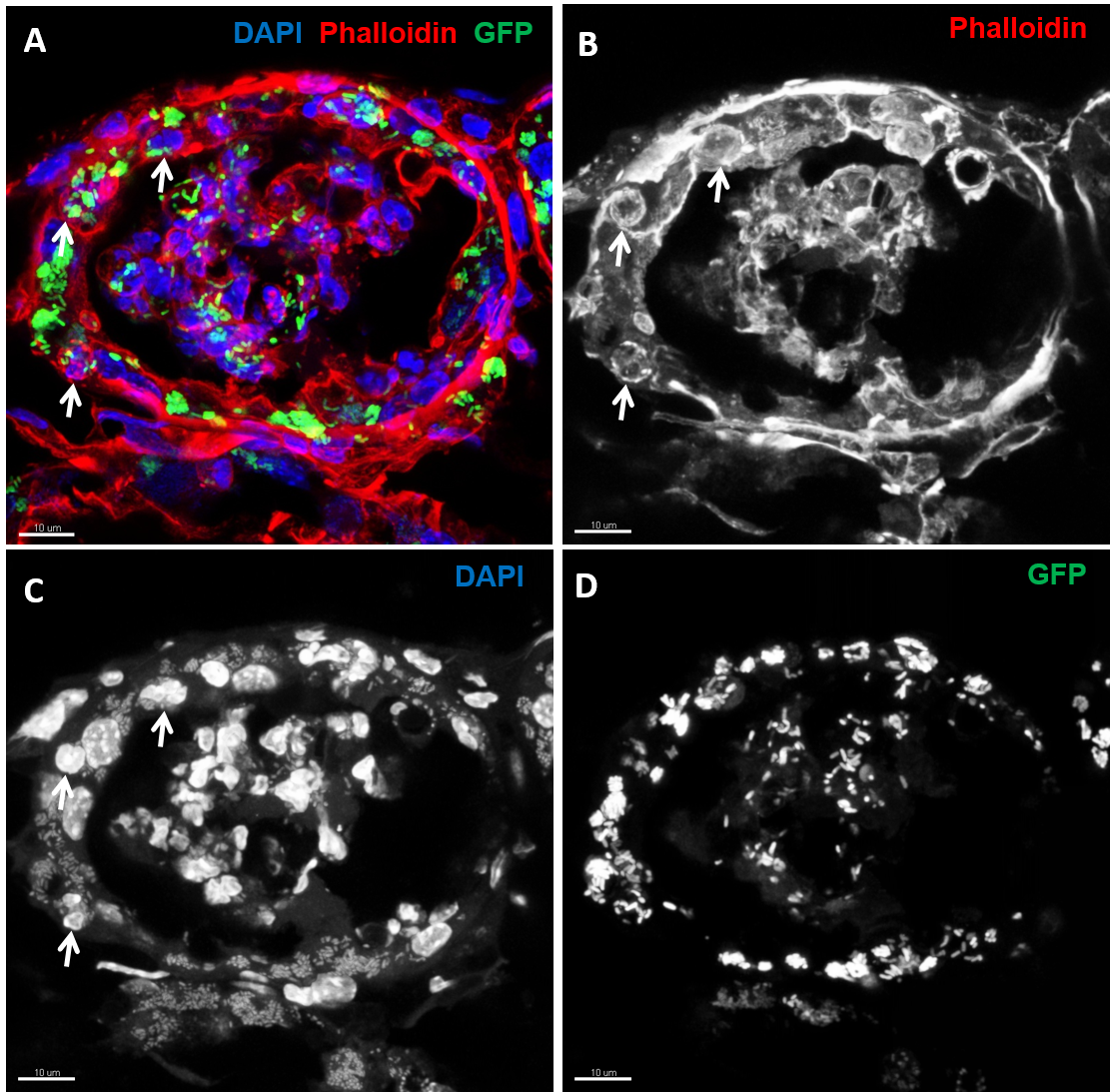

Supplement: Supplementary file 1 — Additional file 1. Fluorescence channels comprising Figure 3A. Mammary gland cryosections stained with DAPI (blue) and phalloidin-TRITC (red) and GFP-expressing MPEC bacteria (A). Confocal microscopy showing a single Z-stack of merged image (A) and separate channels (B–D) in black and white. Scale bars 10 µm. [file 13567_2019_676_MOESM1_ESM.tif]

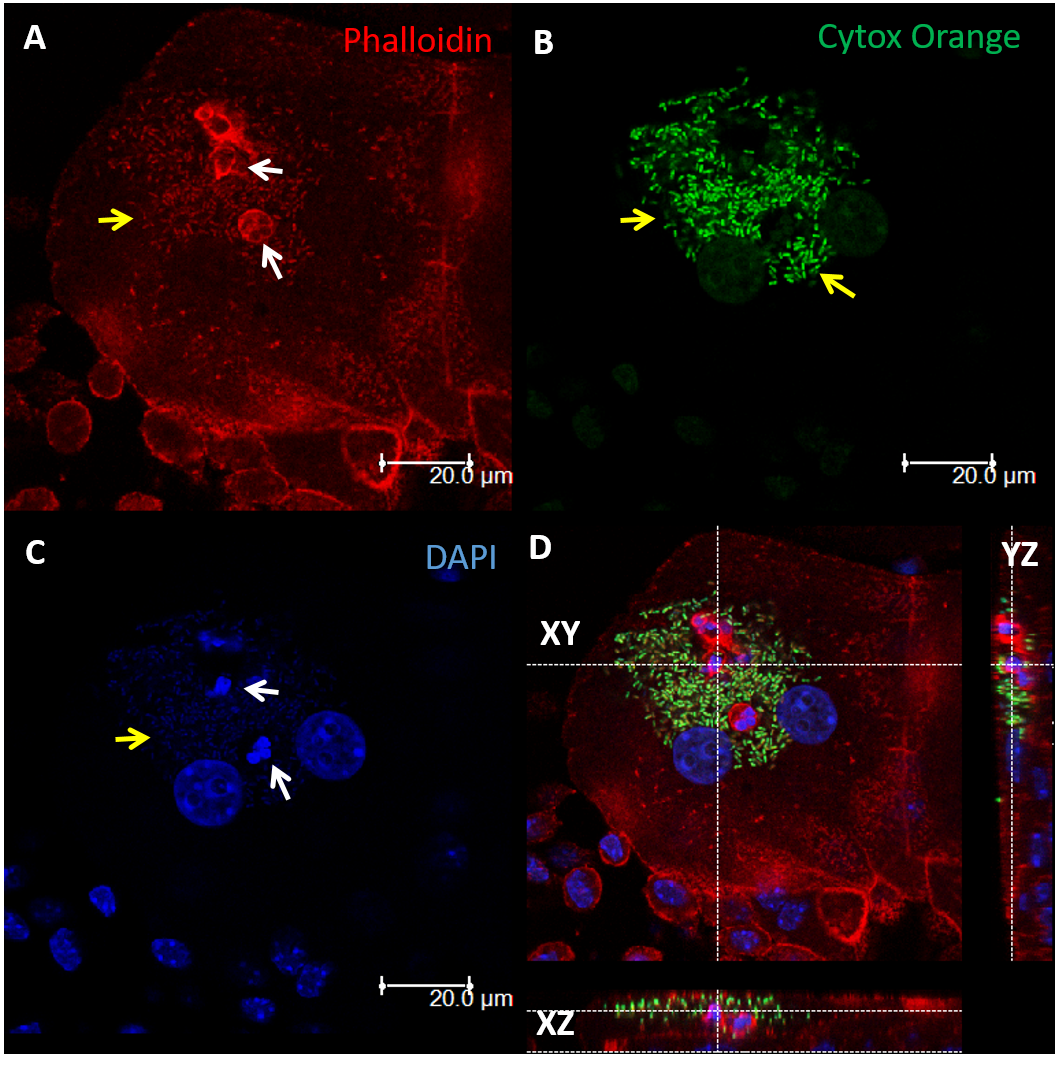

Supplement: Supplementary file 2 — Additional file 2. Bacterial communities are associated with intracellular neutrophils in urinary bladder transitional epithelial cells. Female C57BL/6 mice were challenged by intra-urethral inoculation with 107 CFUs human urinary pathogenic E. coli strain UTI89 and its bladder was harvested 24 h after infection. Whole mounts of urinary bladder were stained with phalloidin-TRITC (A), cytox orange (B), DAPI (C). Confocal laser microscopy demonstrates a large aggregation of intracellular bacterial community (IBC) in superficial umbrella bladder epithelial cell (yellow arrows in A, B) and intraepithelial neutrophil (white arrows in A and C). Composite image is presented in D. The xy image is on the plan indicated by the horizontal and vertical dashed lines shown in the xz and yz images, respectively. Scale bars 20 µm (A–D). [file 13567_2019_676_MOESM2_ESM.tif]

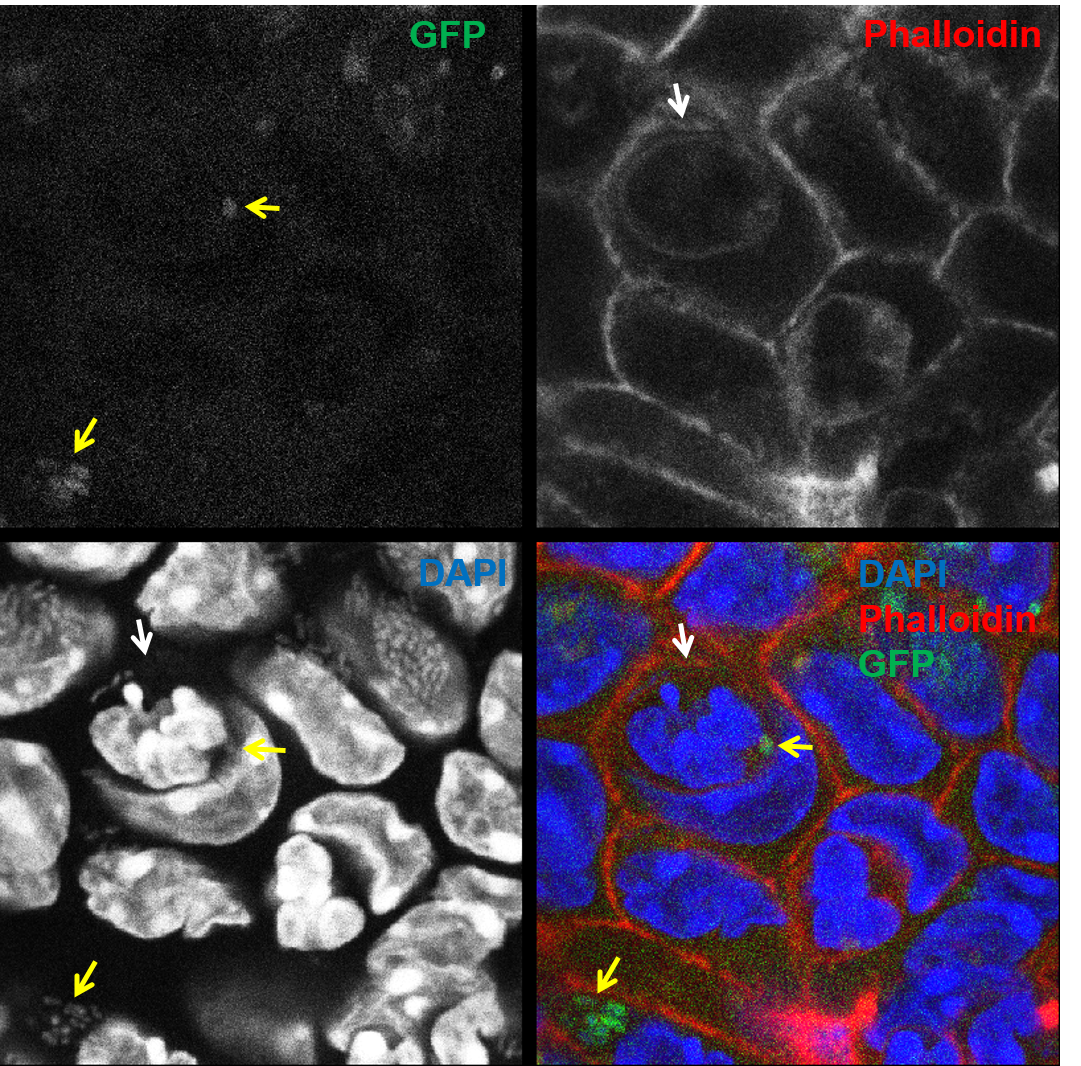

Supplement: Supplementary file 3 — Additional file 3. Bacterial communities are associated with intracellular neutrophils in gall bladder mucosal epithelial cells. Fluorescence channels comprising Figure 4B. Female C57BL/6 mice were challenged by injection of 105 CFUs of Salmonella enterica serovar Typhimurium/PoxyS-gfp strain SL1344 into the gall bladder which was harvested 24 hours after infection. Whole mounts of gall bladder were stained with DAPI (C, D) and phalloidin-TRITC (B and D). Confocal laser microscopy demonstrates GFP-expressing bacteria in gall bladder epithelial cells (yellow arrows in A and C, D) and intraepithelial neutrophils (white arrows in B–D). Composite image is presented in D. Original magnification X63 (A, B). [file 13567_2019_676_MOESM3_ESM.tif]

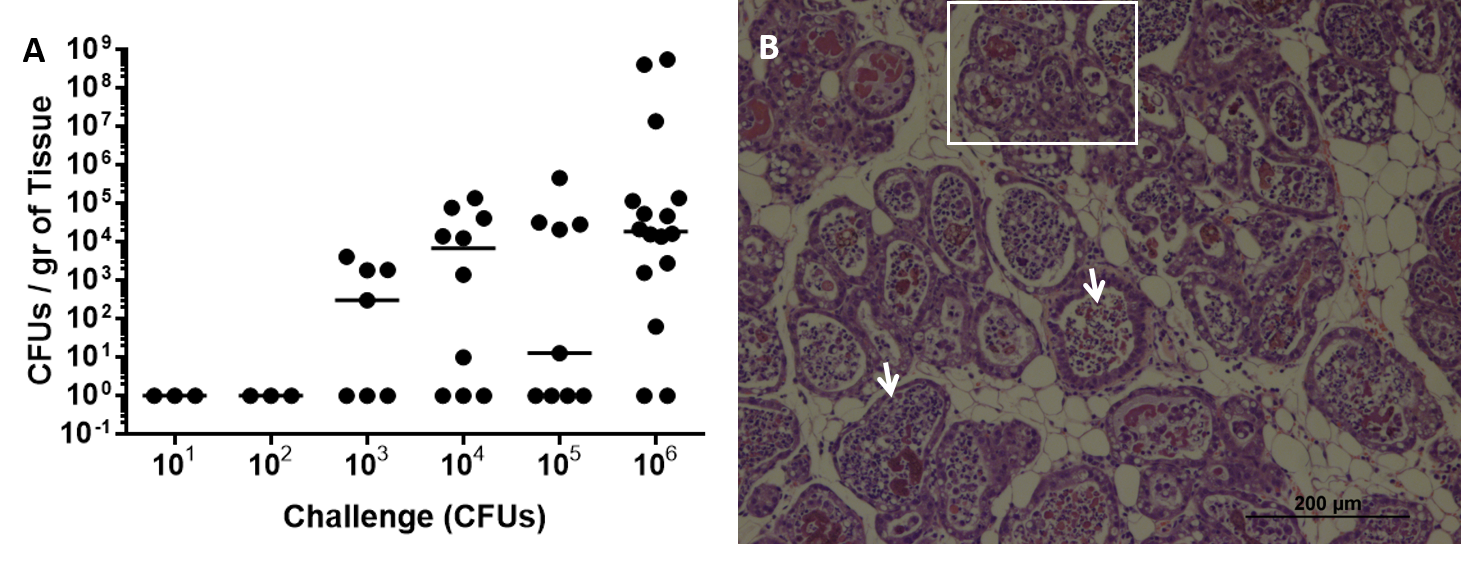

Supplement: Supplementary file 4 — Additional file 4. E. coli DH5α is not mammary pathogenic and does not replicate in the mammary gland following intramammary challenge. A, Mammary bacterial burden 24 hours after intramammary challenge with 101 to 106 viable E. coli DH5α into wild-type C57BL/6 mice are shown. Each symbol represents one gland and all bars represent the median. Medians CFU/gr were analyzed using One-Sample Wilcoxon Signed Rank Test and the null hypothesis was median of CFUs/gr equals to the challenge dose (101 to 106), none of which were statistically significant. H&E staining of formalin-fixed mammary tissues (B–D). Mammary gland with massive recruitment of neutrophils into the alveoli (white arrows in B) 24 h after infusion with 106 viable E. coli DH5α. This is better seen in Figure 5A (white arrow), which is an enlargement of the boxed area in C. Scale bar 200 µm (B). [file 13567_2019_676_MOESM4_ESM.tif]

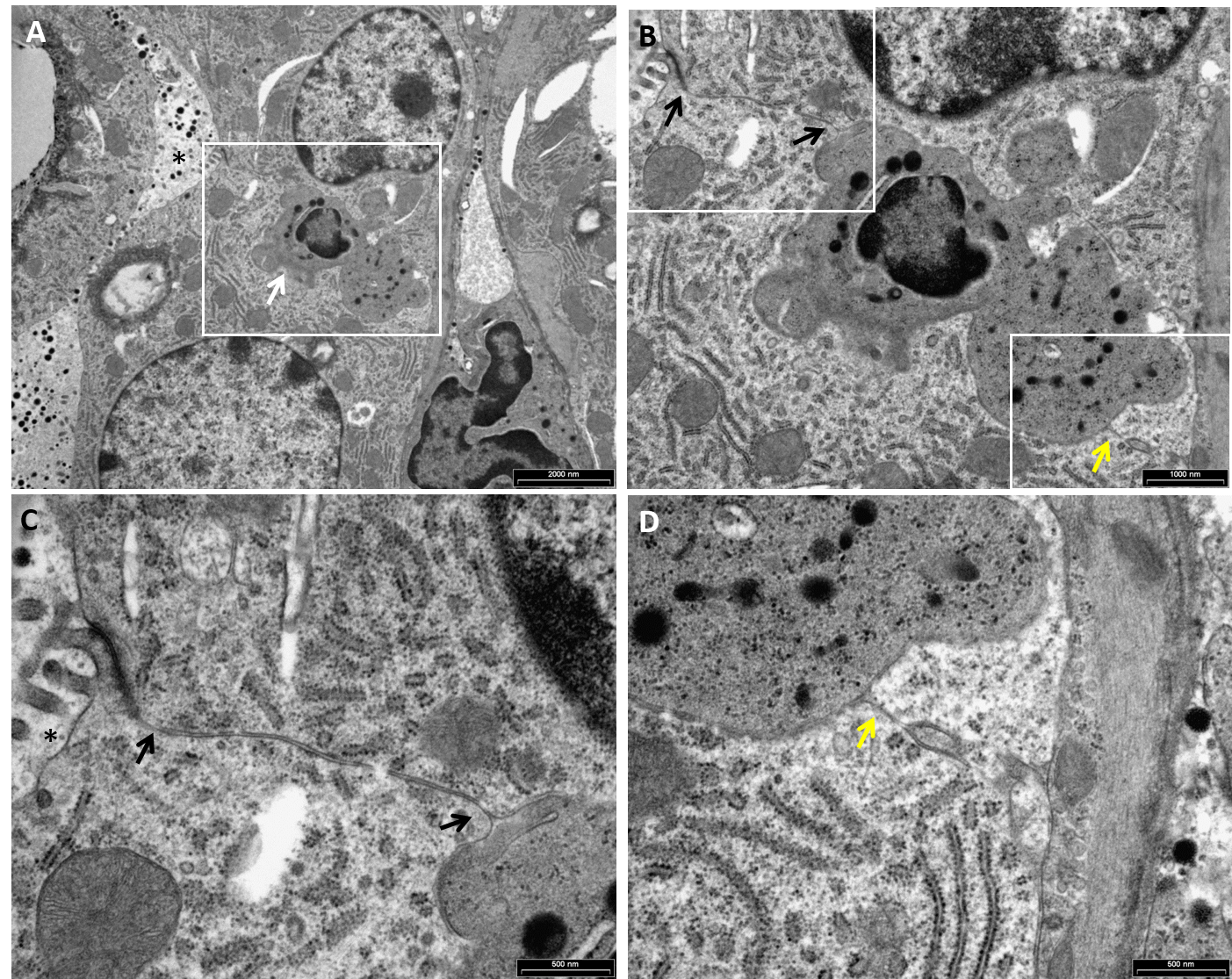

Supplement: Supplementary file 5 — Additional file 5. Live neutrophil (white arrow in A) in mammary epithelial cell enclosed in a double membrane compartment tethered to epithelial cell junctional complex. Lactating C57BL/6 mice where infused with 106 CFUs of viable E. coli DH5α bacteria. Transmission electron microscopy imaging of mammary tissues 24 hours after challenge. Boxed areas in A is enlarged in B and boxed areas in B are enlarged in C, D). Tethering of neutrophil to epithelial junctional complex is visible (black arrows in B, D). Microvilli and milk space (black asterisk * in C) are visible adjacent to the epithelial cell junctional complex (Black arrow in C). Tethering of double membrane to the basolateral membrane of host epithelial cell is also visible (yellow arrows in B and D). Scale bars 2000 nm (A), 1000 nm (B), and 500 nm (C, D). [file 13567_2019_676_MOESM5_ESM.tif]

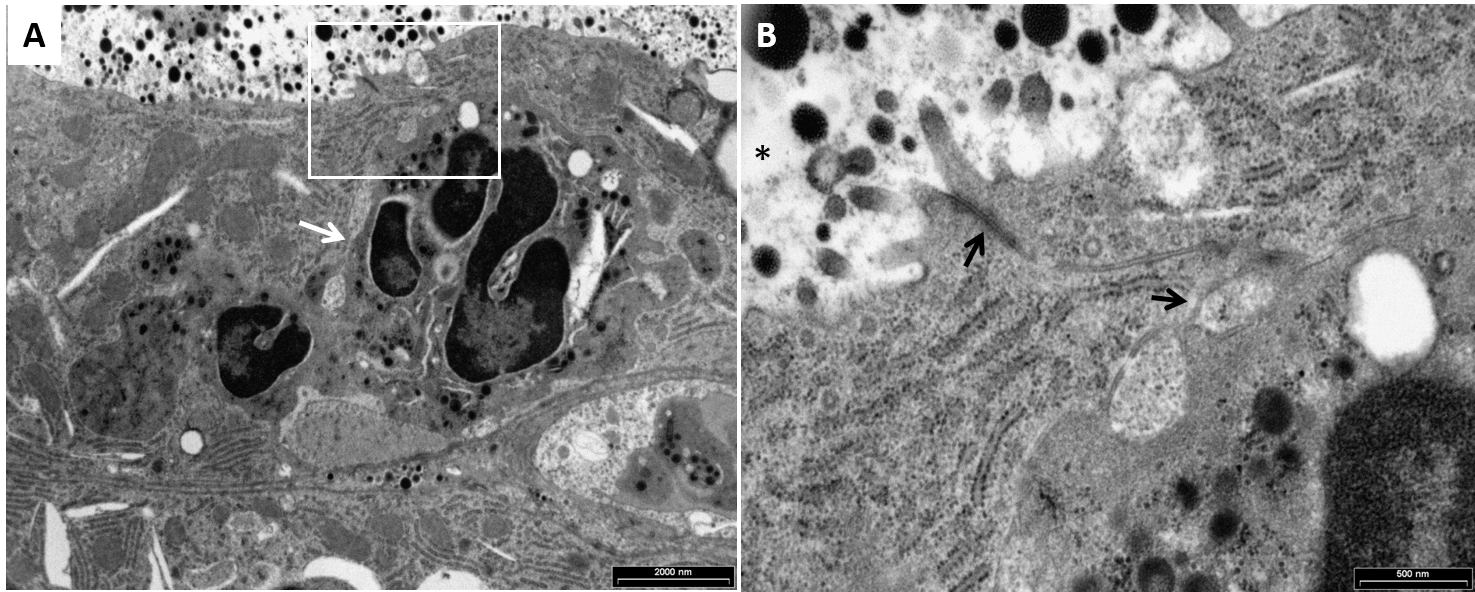

Supplement: Supplementary file 6 — Additional file 6. Live neutrophil (white arrow in A) in mammary epithelial cell enclosed in a double membrane compartment tethered to epithelial cell junctional complex (black arrows in B). Lactating C57BL/6 TLR2−/− mice were challenge by approximately 1000 CFUs via the teat canal. Transmission electron microscopy imaging of mammary tissues 24 h after challenge. Boxed area is enlarged in B. Scale bars 2000 nm (A), and 500 nm (B). [file 13567_2019_676_MOESM6_ESM.tif]

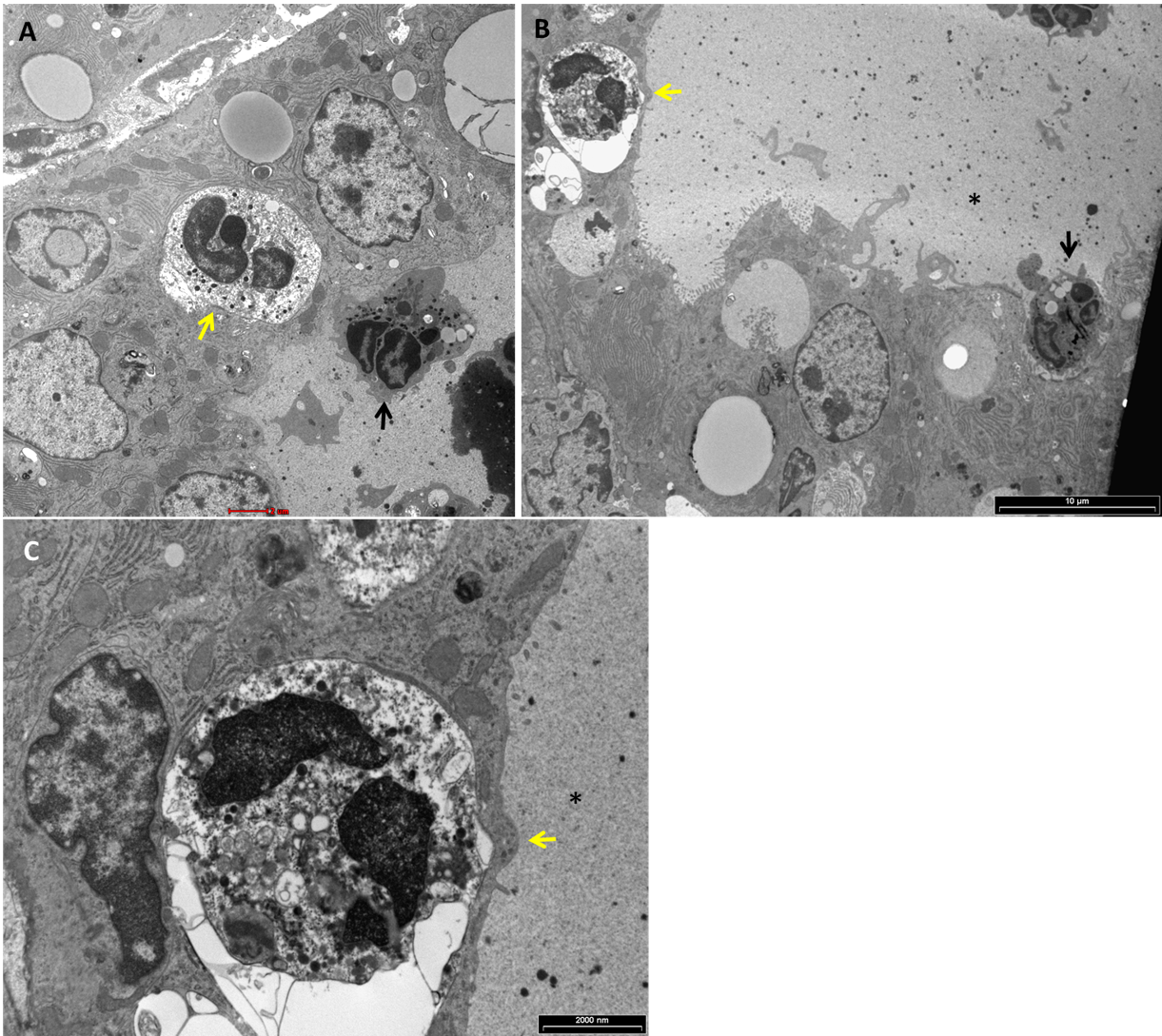

Supplement: Supplementary file 7 — Additional file 7. Entry and apoptosis of neutrophils in mammary epithelium. Lactating C57BL/6 mice where infused with 106 CFUs of viable E. coli DH5α bacteria. Transmission electron microscopy imaging of mammary tissues 24 h after challenge. Live neutrophil adhering to the apical membrane of alveolar epithelial cell (black arrow in A) and commencing the entry process (black arrow in B; see enlarged details in Additional file 8). Early (yellow arrow in A) and late (yellow arrow in B, C) apoptosis of neutrophils are also visible. Scale bars 2 µm (A), 10 µm (B), and 2000 nm (C). [file 13567_2019_676_MOESM7_ESM.tif]

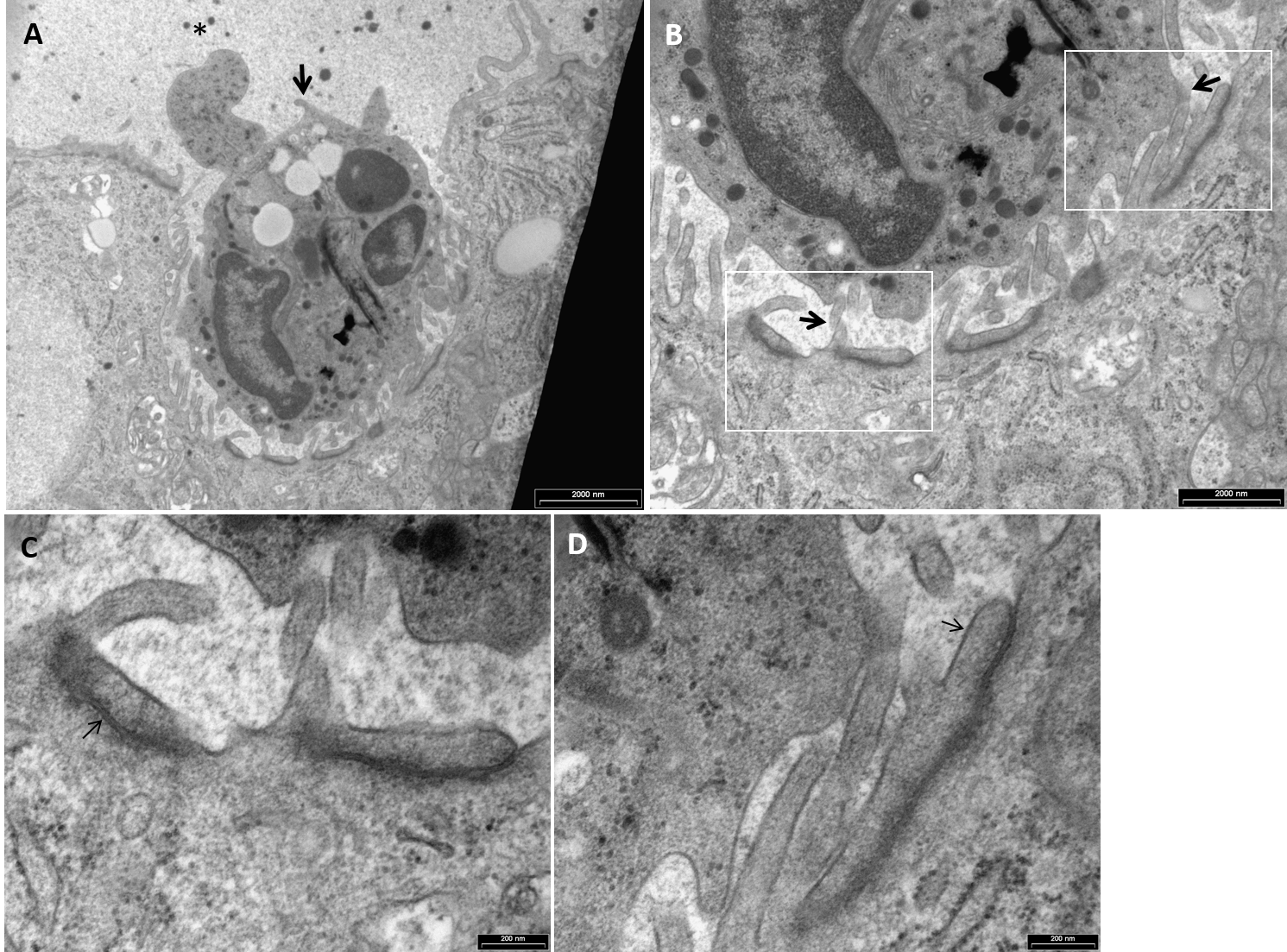

Supplement: Supplementary file 8 — Additional file 8. Commencement of entry by live neutrophil (black arrow in A) interacting with microvilli (black arrows in B-D) on the apical membrane of alveolar epithelial cell. Lactating C57BL/6 mice where infused with 106 CFUs of viable E. coli DH5α bacteria. Transmission electron microscopy imaging of mammary tissues 24 h after challenge. Boxed areas in B are enlarged in C and D. The alveolar milk space in indicated by * in A. Scale bars 2000 nm (A, B), and 200 nm (C, D). [file 13567_2019_676_MOESM8_ESM.tif]

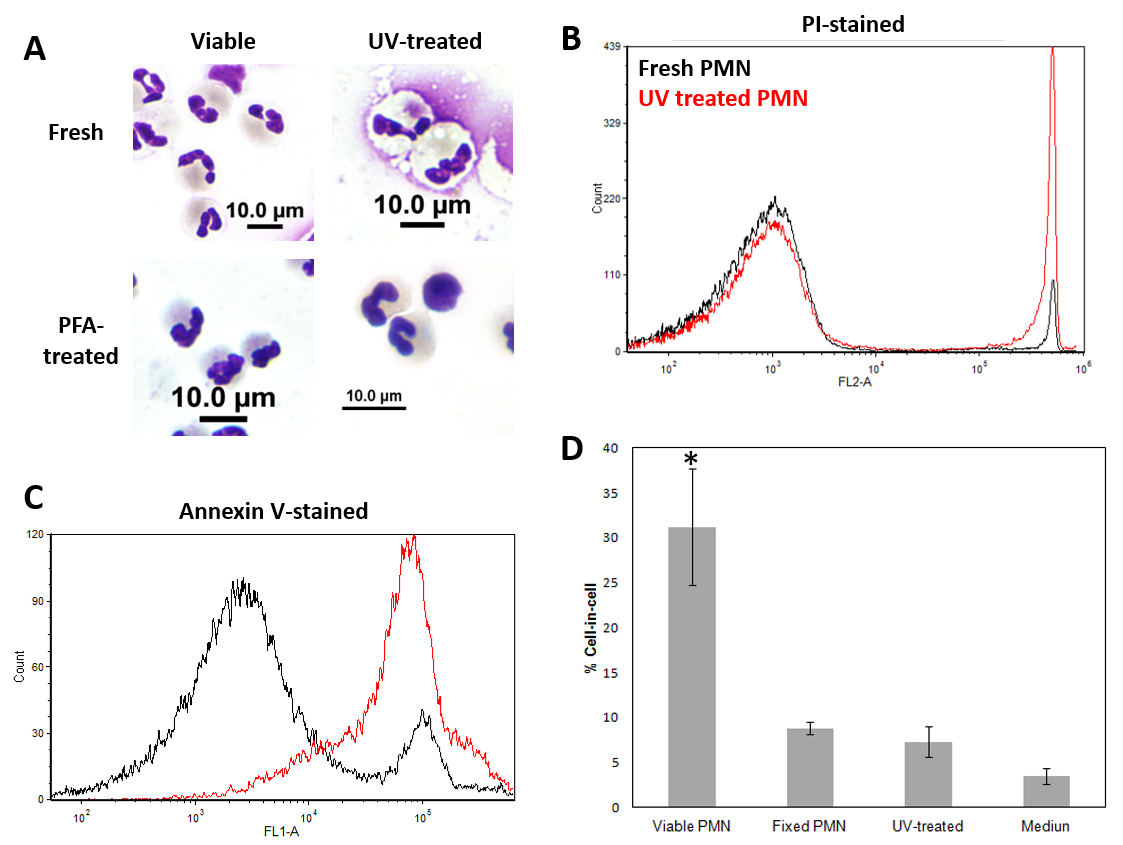

Supplement: Supplementary file 9 — Additional file 9. Viability is essential for entry of neutrophils into mammary epithelial cells. Fresh viable, PFA-fixed and UV-treated apoptotic neutrophils were layered over a monolayer of polar mammary epithelial cell line EPH4 and evaluated microscopically for neutrophil internalization after 24 h of co-culture as described in the materials and methods. Neutrophils were cytospun onto glass slides and stained with Diff Quick for microscopic evaluation (A). Neutrophil viability and apoptosis were quantified using FACS analysis following staining with propidium iodide (PI in B) and Annexin V FITC (C). Mean percentage (± SD) of epithelial cells with internalized cells following co-culture with viable, fixed and UV-treated apoptotic neutrophils is presented in D. Results of a representative experiment out of three. Mean % cell-in-cell following co-culture with viable neutrophils was compared by unpaired t test using GraphPad Prism 6 (GraphPad Software, Inc.) and was significantly different from all other groups; *P < 0.005. Scale bars 10 µm (A). [file 13567_2019_676_MOESM9_ESM.tif]

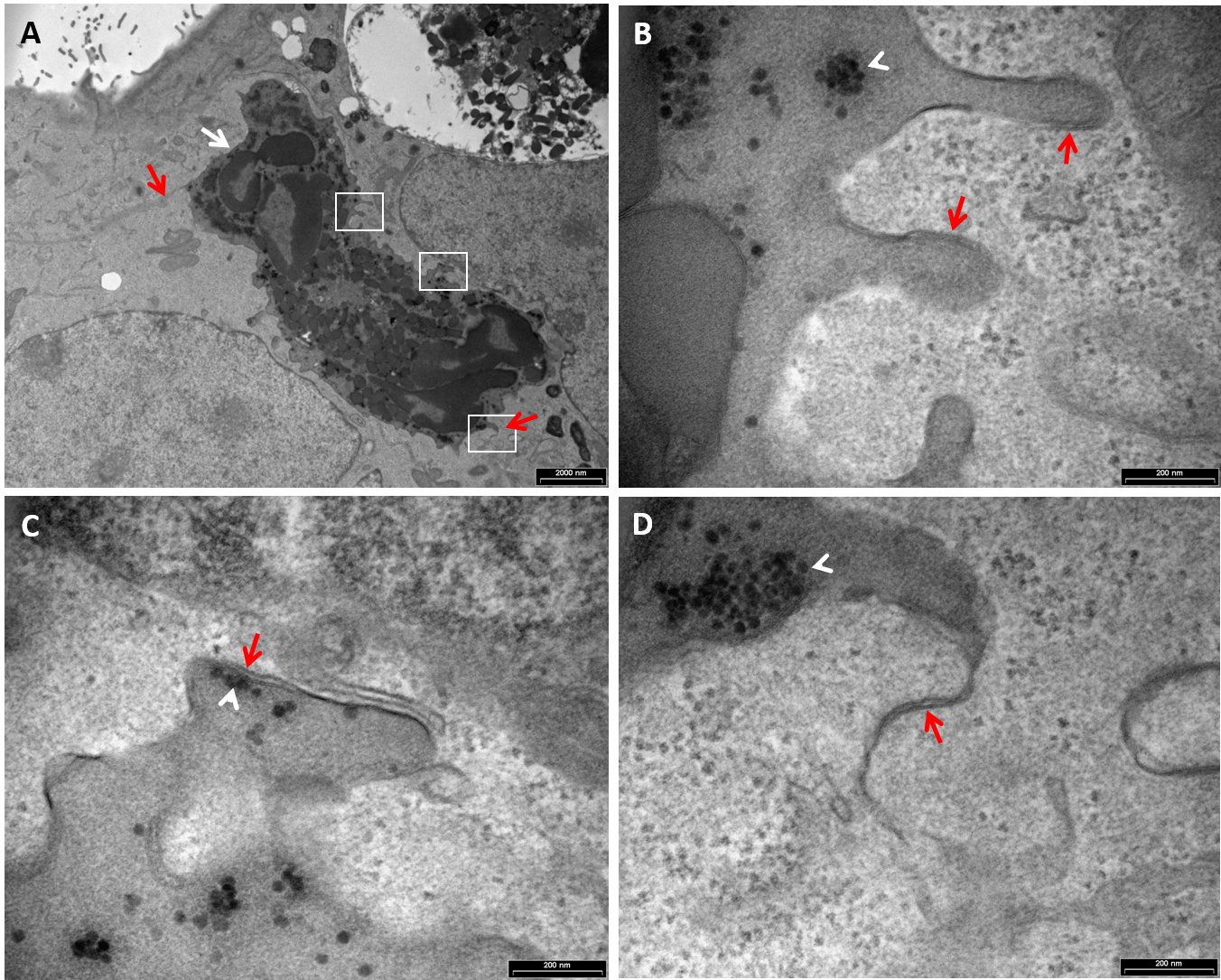

Supplement: Supplementary file 10 — Additional file 10. Intracellular live neutrophil is enclosed in double membrane compartment (enlarged details of Figure 9A). Isolated fresh bovine blood neutrophils were placed on a monolayer of human urinary epithelial cell line 5637 grown on glass cover slides in 24 wells culture plate. After 12 h cells were fixed with PFA and imaged using TEM. Scale bars 2000 nm (A) and 200 nm (B–D). Top, middle and bottom boxed areas in A are enlarged in B, C and D, respectively. Live neutrophil (white arrow in A) is enclosed in a double membrane compartment (red arrows in A–D). Neutrophil is clearly identified by its multilobulated nucleus and the presence of typical glycogen granules in the cytoplasm (white arrow heads in C, D). [file 13567_2019_676_MOESM10_ESM.tif]
